# Hypoculoside, a sphingoid base-like compound from *Acremonium* disrupts the membrane integrity of yeast cells

**DOI:** 10.1038/s41598-018-35979-z

**Published:** 2019-01-24

**Authors:** Mohammad Alfatah, Jin Huei Wong, Choy Eng Nge, Kiat Whye Kong, Kia Ngee Low, Chung Yan Leong, Sharon Crasta, Madhaiyan Munusamy, Amanda Mun Leng Chang, Shawn Hoon, Siew Bee Ng, Yoganathan Kanagasundaram, Prakash Arumugam

**Affiliations:** 1 Bioinformatics Institute, 30 Biopolis Street, #07-01, Matrix, 138671 Singapore; 2 Molecular Engineering Laboratory, 61 Biopolis Drive, #03-12, Proteos, 13867 Singapore

## Abstract

We have isolated Hypoculoside, a new glycosidic amino alcohol lipid from the fungus *Acremonium* sp. F2434 belonging to the order *Hypocreales* and determined its structure by 2D-NMR (Nuclear Magnetic Resonance) spectroscopy. Hypoculoside has antifungal, antibacterial and cytotoxic activities. Homozygous profiling (HOP) of hypoculoside in *Saccharomyces cerevisiae* (budding yeast) revealed that several mutants defective in vesicular trafficking and vacuolar protein transport are sensitive to hypoculoside. Staining of budding yeast cells with the styryl dye FM4-64 indicated that hypoculoside damaged the vacuolar structure. Furthermore, the propidium iodide (PI) uptake assay showed that hypoculoside disrupted the plasma membrane integrity of budding yeast cells. Interestingly, the glycosidic moiety of hypoculoside is required for its deleterious effect on growth, vacuoles and plasma membrane of budding yeast cells.

## Introduction

**Figure 1:**
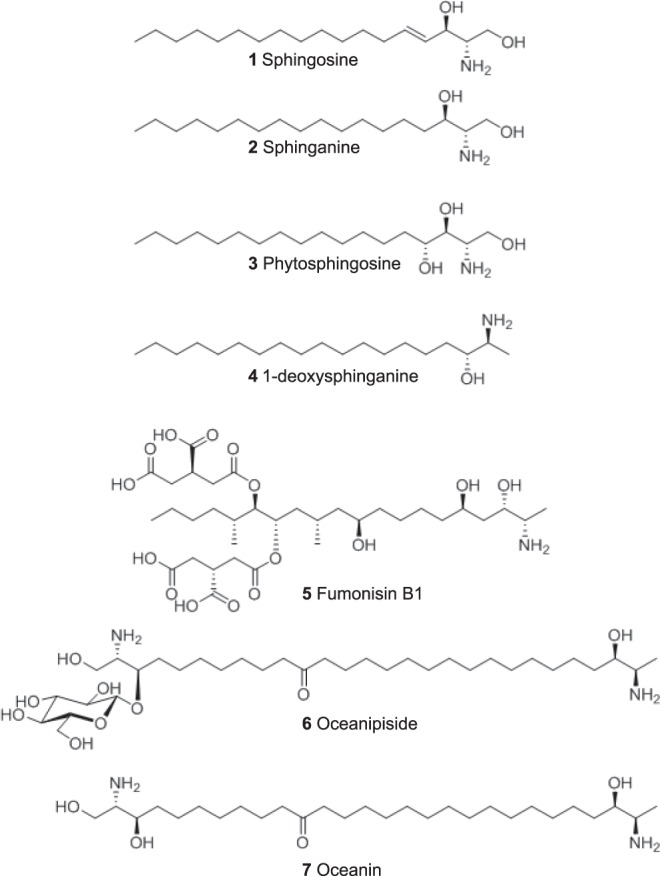
Diversity of sphingoid-bases in nature. Structures of Sphingosine (**1**), Sphinganine/Dihydrosphingosine (**2**), Phytosphingosine (**3**), 1-deoxysphinganine (**4**) Fumonisin B1 (**5**), Oceanipiside (**6**) and Oceanin (**7**) are depicted.

Sphingoid bases (also referred to as sphingosines or long-chain bases) are long-chain aliphatic amino alcohols that serve as precursors of a variety of sphingolipids. Sphingosine specifically refers to (2S,3R,4E)-2-amino-4-octadecen-1,3-diol, a C18 aliphatic chain with an amine group at C2, hydroxyl groups at C1 and C3 and a double bond at C4 (compound **1**: Fig. 1). N-acylation of sphingosine by fatty acids results in the formation of a ceramide. Complex sphingolipids are generated by addition of various head groups to ceramide. Sphingomyelins are formed by esterification of the C1 hydroxyl group of ceramide with charged groups such as ethanolamine and choline. Attachment of single sugars (glucose or galactose) and multiple sugars (containing sialic acid) to the C1 hydroxyl group of ceramide generates cerebrosides and gangliosides, respectively. Several other modifications of ceramides have been identified thus resulting in a diverse family of sphingolipids^1^. Sphingolipids not only play crucial roles in modulating membrane structure and fluidity but also act as intracellular second messengers and regulate growth and differentiation in eukaryotes^2^.

Like sphingolipids, the sphingoid bases themselves display considerable structural diversity in different organisms^3^. Variations could be due to length of the carbon chain, the presence of double bonds or hydroxyl groups or branched side chains at different positions along the hydrocarbon chain. Sphinganine, also called as dihydrosphingosine, (compound **2**: Fig. 1), is a long-chain base that lacks the double bond at C4 present in sphingosine and is found in several organisms^3^. Another long-chain base found in yeasts and some plants is phytosphingosine (compound **3**: Fig. 1) that contains a hydroxyl group attached to C4 of sphinganine^3^.

Underivatized sphingoid bases exhibit a wide range of bioactivities. Sphingosine can be phosphorylated to form sphingosine1-phosphate, a signaling lipid involved in regulation of cell growth and apoptosis in mammalian cells^4^. Sphingoid bases display biological activities such as anti-oxidation, anti-tumor and inhibition of keratinocyte differentiation^5–7^. Sphingoid bases from sea cucumber induce apoptosis in human hepatoma HepG2 cells^8^. Sphingoid bases from plants decrease the levels of TNF-α and IL-8 in human endothelial cells^9^.

Deoxysphingoid bases that lack the hydroxyl group at C1 in sphingosine have also been observed in nature. For example, the clam *Spisula polynyma* produces spisulosine or 1-deoxysphinganine (compound **4**: Fig. 1) which has potent activity against cancer cell lines^10^. Another class of deoxysphingoid-like bases is the fumonisins produced by the pathogenic fungus *Fusarium verticillioides*. Fumonisin B1, the most potent amongst the fumonisins, is a 20-carbon chain compound with an amine group at C2, methyl groups at C12 and C15, hydroxyl groups at C3, C4 and C9, and tricarballylic ester moieties at C13 and C14 (compound **5**: Fig. 1). Fumonisin B1 is synthesized by the FB biosynthetic gene cluster which consists of 17 transcriptionally coregulated genes^11–14^. Owing to its structural similarity to long-chain bases, fumonisin B1 blocks ceramide biosynthesis by inhibiting the N-acylation of sphingosine^15^.

An interesting class of deoxysphingoid bases is the α, ω-bi-functionalized amino alcohols that contain two sphingoid-like bases attached tail to tail. One example is the antifungal oceanin, which is the aglycone derivative of oceanapiside (compound **6**: Fig. 1) isolated from the marine sponge *Oceanapia phillipensis*^16^. Oceanin is a 28-carbon chain compound with three hydroxyl groups and two amino groups (compound **7**: Fig. 1). Oceanin is considerably more toxic to fungi than its glycosidic variant oceanapiside^16^.

**Figure 2:**
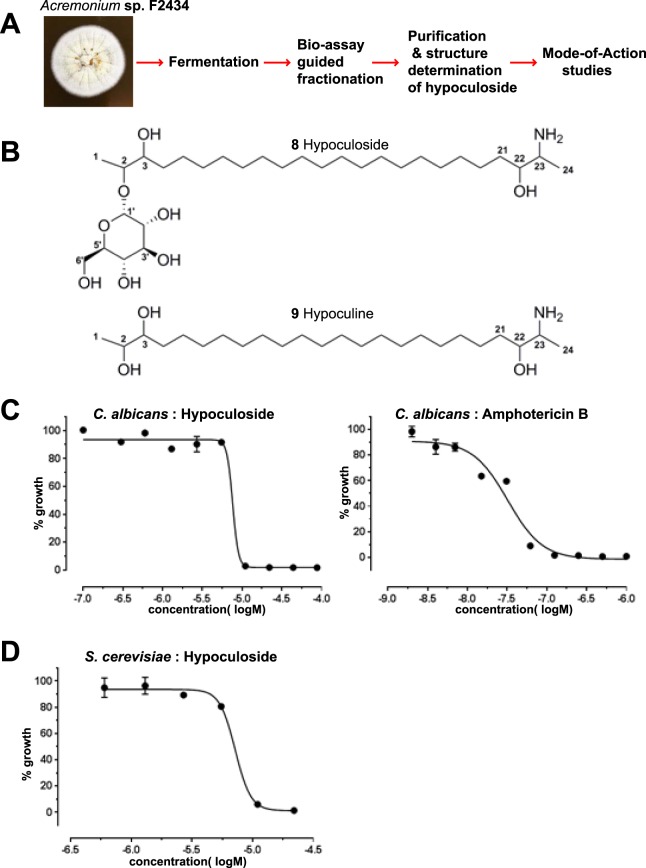
Hypoculoside has antifungal activity. (**A**) Workflow used in the purification and analysis of hypoculoside from *Acremonium* sp. F2434. (**B**) Structure of hypoculoside (**8**) and its aglycone derivative hypoculine (**9**). (**C**) Logarithmically growing *Candida albicans* cells were exposed to hypoculoside and amphotericin B at various concentrations in triplicates in a 96-well microplate. Growth of the cells was quantified by recording the absorbance at 600 nm after 24 hours. Growth (normalized with respect to DMSO-treated cells) is plotted against log of concentration of the compound. A picture of the microplate after 24 hours of incubation at 30 °C is shown in Supplementary Fig. S2A. (**D**) Effect of hypoculoside on the growth of *Saccharomyces cerevisiae* cells grown in YPD medium was analyzed in the similar way as for *Candida albicans* described above in C.

Due to the continuing emergence of multi-drug resistant microbes and a constant need for new antimicrobials, we are mining a comprehensive Natural Organism Library^17^ for the presence of novel bioactive compounds and determining their mode of action (Fig. 2A). In this paper, we report the isolation, structure determination and Mode-of-Action studies of a new deoxysphingoid derivative called hypoculoside from the fungus *Acremonium* sp. F2434. Hypoculoside has antifungal activity against both *Candida albicans* and *Saccharomyces cerevisiae*. Chemogenomic profiling of hypoculoside in *Saccharomyces cerevisiae* indicated that the vesicular trafficking machinery confers resistance to the compound. We show that hypoculoside disrupts the vacuolar structure and plasma membrane permeability of yeast cells. We suggest that the vesicular trafficking mutants having defective vacuoles are more sensitive to the inhibitory action of hypoculoside.

## Results

### Isolation and structure determination of hypoculoside and aglycone hypoculine

In a screen for natural compounds with antifungal activity, we obtained an active methanolic extract from the fungal strain *Acremonium* sp. F2434 (Fig. 2A) that inhibited the growth of *Candida albicans* in the whole cell activity assay^18^. Bioassay-guided fractionation^18^ led to the isolation of a hitherto unreported glycosidic amino hydroxy lipid, hypoculoside (compound **8**: Fig. 2B), possessing a 24-membered linear chain. Hypoculoside (**8**) was assigned a molecular formula of C_30_H_61_NO_8_ based on the HRESIMS m/z at 564.4474 [M + H]^+^ (calcd for C_30_H_61_NO_8_+H, 564.4475), which is consistent with one degree of unsaturation. Hypoculoside (**8**) did not show any absorption above λ_210 nm_ in its UV spectrum, indicating the absence of conjugation. Its ^1^H, ^13^C NMR and multiplicity-edited gradient Heteronuclear Single Quantum Coherence (HSQC) spectroscopy indicated the presence of two terminal methyl groups (δ_H_ 1.12,1.20 and δ_C_ 12.1, 12.2; Table 1), one oxygenated methylene, eight oxygenated methines and one methine with amino group. The remaining carbon and hydrogen atoms were assigned to long alkyl chain based on the overlapping signals for methylene protons (δ_H_ 1.26–1.49) and the corresponding carbon signals (δ_C_ 27.0–34.1). Analysis of ^1^H NMR, ^13^C NMR, COSY, HSQC and HMBC spectra of hypoculoside aglycone moiety showed the presence of two terminal sequences as -CH(OH)CH(OR)CH_3_ and -CH(OH)CH(NH_2_)CH_3_ spin systems of a long linear chain amino hydroxy lipid. The signals observed at δ_C_ 96.7 and δ_H_ 4.94 (d, *J* = 3.9 Hz), δ_C_ 73.5 and δ_H_ 3.38 (dd, *J* = 9.7, 3.9 Hz), δ_C_ 75.2 and δ_H_ 3.62 (m), δ_C_ 74.0 and δ_H_ 3.62 (m), δ_C_ 71.9 and δ_H_ 3.27 (m), δ_C_ 62.7 and δ_H_ 3.64 (m), 3.78 (m) suggested the presence of a sugar moiety. In addition, the LC-MS/MS analysis of m/z 564 revealed a major fragment ion at m/z 402 (Supplementary Fig. S1), which corresponded to the neutral loss of a glucosyl unit from hypoculoside. Treatment of hypoculoside with 4 M HCl followed with purification by C-18 column chromatography generated the aglycone unit, hypoculine (Compound **9**: Fig. 2B) and sugar. The sugar was identified as D-glucose by HPLC (Shodex KS-G and KS-801 columns with mobile phase 100% water) in comparison with an authentic standard and has the α-configuration at the anomeric carbon as shown by the coupling constant for the anomeric proton at δ_H_ 4.94 (d, *J* = 3.9 Hz). Site of linkage of the D-glucose group in hypoculoside (**8**) was determined on the basis of HMBC correlations from H-2 to C-1′, from H-1′ to C-2 and C-3′, thereby establishing the D-glucose moiety was linked to C-2. The remainder of the ^1^H NMR signals for hypoculoside (**8**) could be attributed to long methylene chains (δ_H_ 1.26–1.49, m, δ_C_ 27.0–34.1). The aglycone (**9**) molecular formula was C_24_H_51_NO_3_, as deduced from HRESIMS m/z at 402.3950 [M + H]^+^ (calcd for C_24_H_51_NO_3_+H, 402.3947), indicating that it contains 24 carbons. The ^1^H, ^13^C, COSY, edited HSQC and HMBC of hypoculoside confirmed the presence the of the three moieties -CH(OH)CH(OR)CH_3_, -CH(OH)CH(NH_2_)CH_3_ and long methylene chains. We thus established the structure of hypoculoside (**8**), as an unusual glycosylated deoxysphingoid-like compound. Structures of hypoculoside (**8**) and hypoculine (**9**) are depicted in Fig. 2B.

**Table 1:** NMR spectral data^a^ of hypoculoside (**8**) and hypoculine (**9**).

Position	8		9	
	^13^C^b^	^1^H^c^, mult. (*J* = Hz)	^13^C^b^	^1^H^c^, mult. (*J* = Hz)
1	12.1	1.12, d (6.5)	18.4	1.14, d (6.5)
2	75.5	3.74, m	71.8	3.68, m
3	75.4	3.69, m	76.7	3.35, m
4	34.1	1.44, m, 1.44, m	33.8	1.34, m, 1.54, m
5	27.0	1.31, m, 1.49, m	27.0	1.33, m, 1.55, m
6–19	30.6–30.8	1.26–1.34, m	30.6–30.8	1.28, m
20	27.2	1.32, m, 1.32, m	27.1	1.33, m, 1.33, m
21	34.0	1.43, m, 1.43, m	34.0	1.44, m
22	71.8	3.68, m	71.7	3.69 m
23	52.6	3.25, m	52.6	3.25, ddd (10, 6.7, 3)
24	12.2	1.20, d (6.5)	12.2	1.20, d (6.5)
1′	96.7	4.94, d (3.9)		
2′	73.5	3.38, dd (9.7, 3.9)		
3′	75.2^d^	3.62, m		
4′	74.0^d^	3.62, m		
5′	71.9	3.27, m		
6′	62.7	3.64, m, 3.78, m		

^a^Assignments based on COSY, HSQCED and HMBC. ^b^(**8**) and (**9**) were recorded at 100 MHz with CD_3_OD as internal standard at δ 49.0 Chemical shifts (δ) in ppm. ^c^(**8**) and (**9**) was recorded at 400 MHz with CD_3_OD as internal standard at δ 3.30. ^d^Assignments are interchangeable. s: singlet; d: doublet. q: quartet; m: multiplet; br; broad.

### Hypoculoside has antifungal, antibacterial and cytotoxic activities

We evaluated the potency of hypoculoside by examining its effect on the growth of *Candida albicans* strain (SC5314) using the Clinical and Laboratory Standards Institute (CLSI) guidelines^19^. We used the well-known antifungal amphotericin B as a positive control. We exposed logarithmically growing *Candida albicans* cells to hypoculoside and amphotericin B at various concentrations and monitored the growth by recording the OD_600 nm_ after 24 hours. Consistent with published data^20^, amphotericin B completely inhibited the growth of *C*. *albicans* cells with an IC_50_ of 32 nM (Fig. 2C and Supplementary Fig. S2A). Hypoculoside completely inhibited the growth of *C*. *albicans* cells with an IC_50_ of 7.6 μM (Fig. 2C and Supplementary Fig. S2A). We also tested the effect of hypoculoside on the growth of the *Saccharomyces cerevisiae* strain (BY4743). Hypoculoside prevented the growth of *Saccharomyces cerevisiae* cells with an IC_50_ of 7.2 μM (Fig. 2D). To test whether hypoculoside has cytocidal or cytostatic activity, we examined the ability of *Saccharomyces cerevisiae* cells to recover following treatment with either hypoculoside (21 µM) or DMSO for 24 h. Unlike DMSO-treated cells, hypoculoside-treated cells were unable to grow after their transfer to hypoculoside-free YPD agar plates indicating that hypoculoside has cytocidal activity (Supplementary Fig. S2B). We found that hypoculoside was inhibitory against the Gram-positive bacterium *Staphylococcus aureus* (IC_50_ = 11.7 µM) but not against Gram-negative bacteria, *Pseudomonas aeruginosa* and *Klebsiella aerogenes* (Supplementary Fig. S2C). Hypoculoside also demonstrated cytotoxicity (IC_50_ = 9–14 µM) against human lung and pancreatic carcinoma cell lines (Supplementary Fig. S2D). These results indicate that hypoculoside has antifungal, antibacterial and cytotoxic activities.

### HOP analysis of hypoculoside

**Figure 3:**
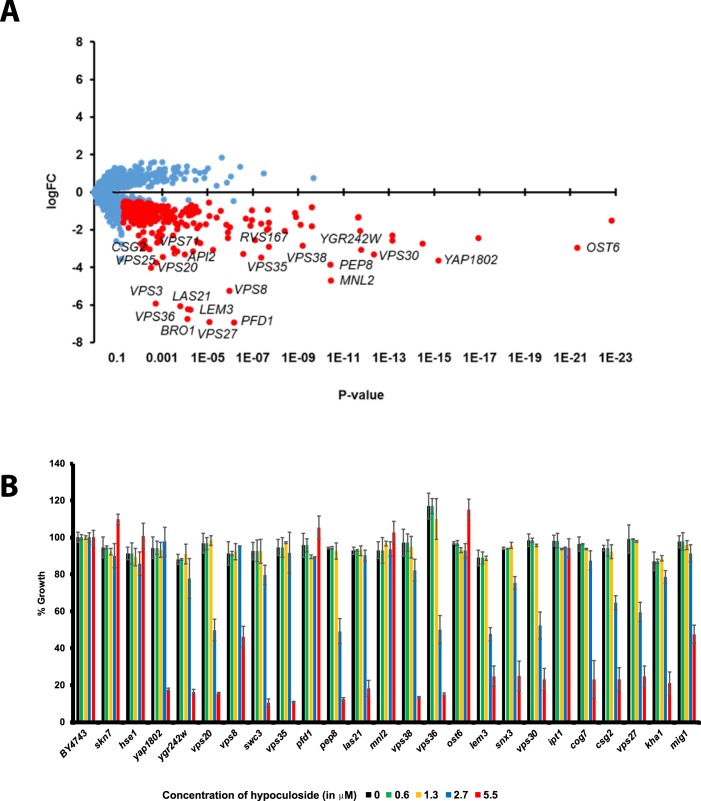
Homozygous profiling of hypoculoside in *Saccharomyces cerevisiae*. (**A**) logFC (Fitness Coefficient) was plotted against P-value for the 3702 mutants that were analyzed by HOP. The more negative the logFC value of a mutant, more sensitive is the mutant to hypoculoside. Mutants that have logFC < −0.5 and P-value < 0.05 are indicated by red dots and the remaining strains are indicated as blue dots. For the sake of clarity, gene names of only a subset of the top hits are indicated in the plot. (**B**) Wild type strain BY4743 and 24 deletion strains that were sensitive to hypoculoside in the HOP assay, were grown in the YPD medium in the presence of either DMSO or hypoculoside at different concentrations (0, 0.6, 1.3, 2.7 and 5.5 µM). Normalized growth at the different concentrations of hypoculoside tested after 24 hours of incubation at 30 °C is plotted for each strain. Representative data from two independent experiments are shown in the Figure.

To gain insights into hypoculoside’s Mode-Of-Action, we performed Homozygous Profiling (HOP) in *Saccharomyces cerevisiae*. This assay uses a collection of bar-coded yeast knockout strains created by the *Saccharomyces cerevisiae* Genome Deletion Project^21^, and can identify non-essential genes that confer either resistance or sensitivity to a compound in a single experiment^22–24^. Pooled mixture of bar-coded KO strains was grown in the absence or presence of hypoculoside for about five generations. Genomic DNA was isolated from the strains and the bar-codes were amplified by PCR and sequenced by Next Generation Sequencing methods. The sequences were then searched against the bar-code database and the Fitness co-efficient of 3702 deletion mutants were obtained (Supplementary Table S1). A negative logFC value indicates sensitivity to the compound and conversely a positive value indicates resistance. The logFC was plotted against P-value (Fig. 3A). Most of the 3702 mutants had logFC values close to 0 but there were some which had a significantly high positive or a negative value.

### Validation of HOP data

To test the validity of our HOP data, we first identified 336 ‘hypoculoside-sensitive’ mutants (Supplementary Table S1) that had at least a 1.4-fold inhibitory effect on their growth rate (i.e. logFC ≤ −0.5) with a P-value ≤ 0.05. From the list of 336 mutants, we chose 24 mutants that represent different Gene Ontology categories (see below) and tested their sensitivity to hypoculoside. Eighteen of the total 24 mutants were sensitive to hypoculoside confirming the HOP data (Fig. 3B). We also tested mutants that were predicted to be resistant to hypoculoside (positive logFC ≥ 0.5 and P-value ≤ 0.05). But none of them were resistant to hypoculoside in comparison to wild type strain in the single-mutant sensitivity assays (data not shown). Hence, we focused on the hypoculoside-sensitive mutants for further analysis.

### Biological Process, Cellular Component and Molecular Function enrichment analysis

**Figure 4:**
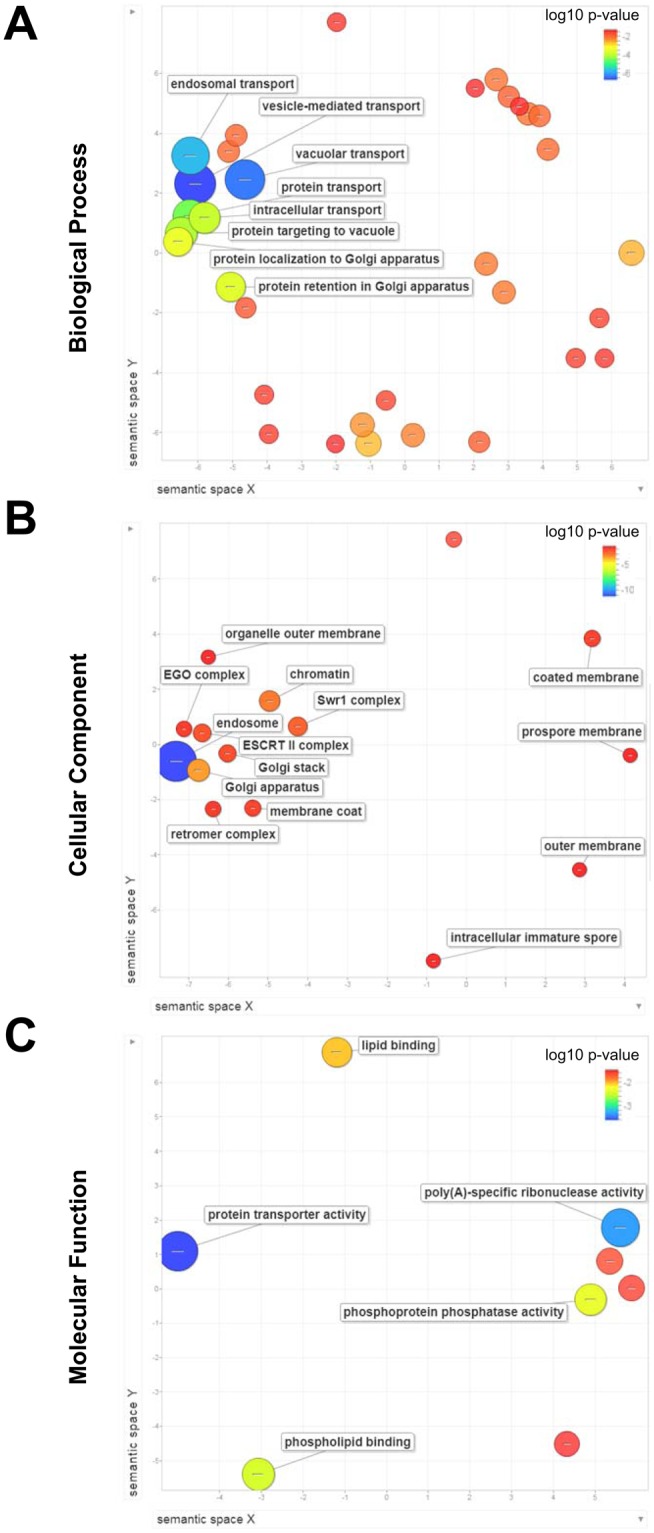
Gene Ontology (GO) analysis of genes conferring resistance to hypoculoside. Enrichment of GO terms among the top 336 genes conferring resistance (logFC value < −0.5 and P-value < 0.05) to hypoculoside was determined by using DAVID in combination with REVIGO. The three plots show the enrichment of GO terms in the following categories: (**A**) Biological Process, (**B**) Cellular Component and (**C**) Molecular Function. For each category, the semantic similarities between the non-redundant GO terms are represented as scatterplots in 2-dimensional space. Related GO terms cluster together in the plot. The p-value for the false discovery rates of each GO term is represented by the colour of the corresponding circle. The GO term frequency in the GO database is represented by the size of the corresponding circle.

To gain insights into hypoculoside’s mode of action, we performed Gene Ontology (GO) analyses of 336 genes that provide resistance to hypoculoside using the online tool DAVID^25,26^. We used REVIGO^27^ to remove the redundant GO enrichment terms and for visualization (Fig. 4). Biological Process enrichment analysis revealed that several hypoculoside-resistance gene products were involved in vesicle-mediated transport (GO:0016192, 44 genes), endosomal transport (GO:0016197,15 genes), vacuolar transport (GO:0007034, 22 genes) and protein localization (GO:0008104, 52 genes) (Fig. 4A and Supplementary Table S1). Cellular Component enrichment analysis indicated that several gene products were localized to the endosomes (GO:0005768, 29 genes) and Golgi apparatus (GO:0005794, 25 genes) (Fig. 4B and Supplementary Table S1). Molecular Function enrichment analysis showed that some of the hypoculoside-resistance gene products were protein transporters (GO:0008565, 12 genes) and phospholipid binding (GO:0005543, 11 genes) (Fig. 4C and Supplementary Table S1). Taken together, our results suggest that hypoculoside exacerbates the growth defect of mutants impaired in vesicle-mediated transport.

### Structure-activity studies with aglycone and commercially available sphingosines

**Figure 5:**
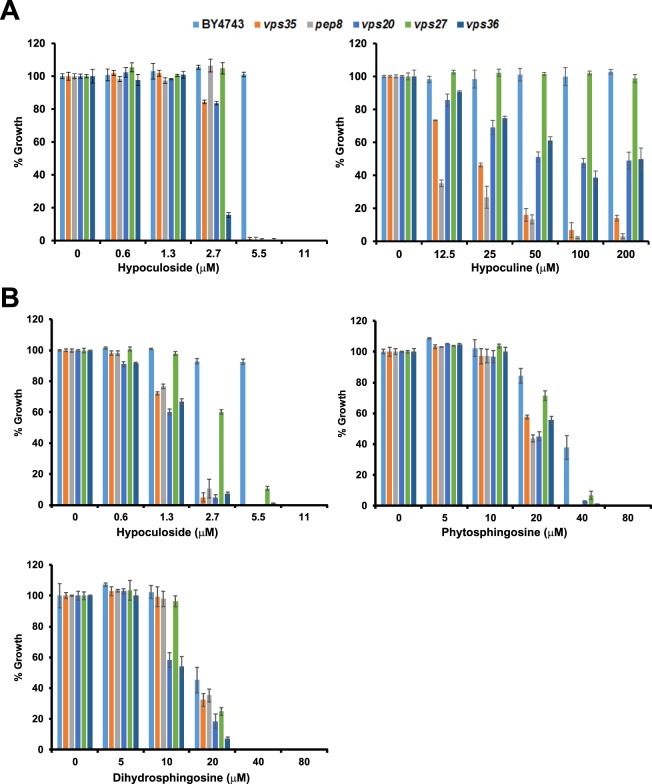
Structure-activity studies of hypoculoside. (**A**) Wild type strain (BY4743), *vps35Δ*, *pep8Δ*, *vps20Δ*, *vps27Δ* and *vps36Δ* strains were grown in YPD medium in the presence of hypoculoside or hypoculine at the indicated concentrations. Normalized growth at the different concentrations of hypoculoside and hypoculine tested after 24 hours of incubation at 30 °C is plotted for each strain. Representative data from three independent experiments are shown in the Figure. (**B**) Wild type strain (BY4743), *vps35Δ*, *pep8Δ*, *vps20Δ*, *vps27Δ* and *vps36Δ* strains were grown in YPD medium in the presence of hypoculoside or phytosphingosine or dihydrosphingosine at the indicated concentrations. Normalized growth at the different concentrations of hypoculoside, phytosphingosine and dihydrosphingosine tested after 24 hours of incubation at 30 °C is plotted for each strain. Representative data from three independent experiments are shown in the Figure.

To understand the basis of hypoculoside’s mode of action, we tested the effect of modifying the structure of hypoculoside on its activity and sensitivity of the various mutants. We firstly tested whether the sugar group is required for hypoculoside’s inhibitory activity. Hypoculoside inhibited the growth of wild type cells at 11 μM and the *vps35*, *pep8*, *vps20*, *vps27* and *vps36* mutants at 5.5 μM. In contrast, wild type and mutants (except for *pep8* and *vps35*) displayed significant growth even at 200 μM hypoculine (Fig. 5A). These results indicate that the sugar moiety is required for the antifungal activity of hypoculoside.

We then compared the toxicity and mutant sensitivity patterns of hypoculoside with two sphingoid bases namely phytosphingosine (PHS) (compound 3: Fig. 1) and dihydrosphingosine (DHS) (compound 2: Fig. 1), which are intermediates in the yeast sphingolipid biosynthetic pathway. PHS and DHS completely inhibited the growth of wild type yeast cells at 40 μM (Fig. 5B) which is about 4-fold higher in comparison to inhibitory concentration of hypoculoside (Fig. 5B). Consistent with earlier data, the *vps35*, *pep8*, *vps20*, *vps27* and *vps36* mutants were completely inhibited at 5.5 µM hypoculoside but the wild type strain was slightly (10%) inhibited. At 20 µM of PHS and DHS, the growth of the 5 hypoculoside-sensitive mutants was lower in comparison to wild type strain (Fig. 5B) suggesting that the toxicity mechanisms of hypoculoside and PHS/DHS are related.

### Hypoculoside does not reverse the myriocin-induced inhibitory effect on sphingolipid biosynthesis

**Figure 6:**
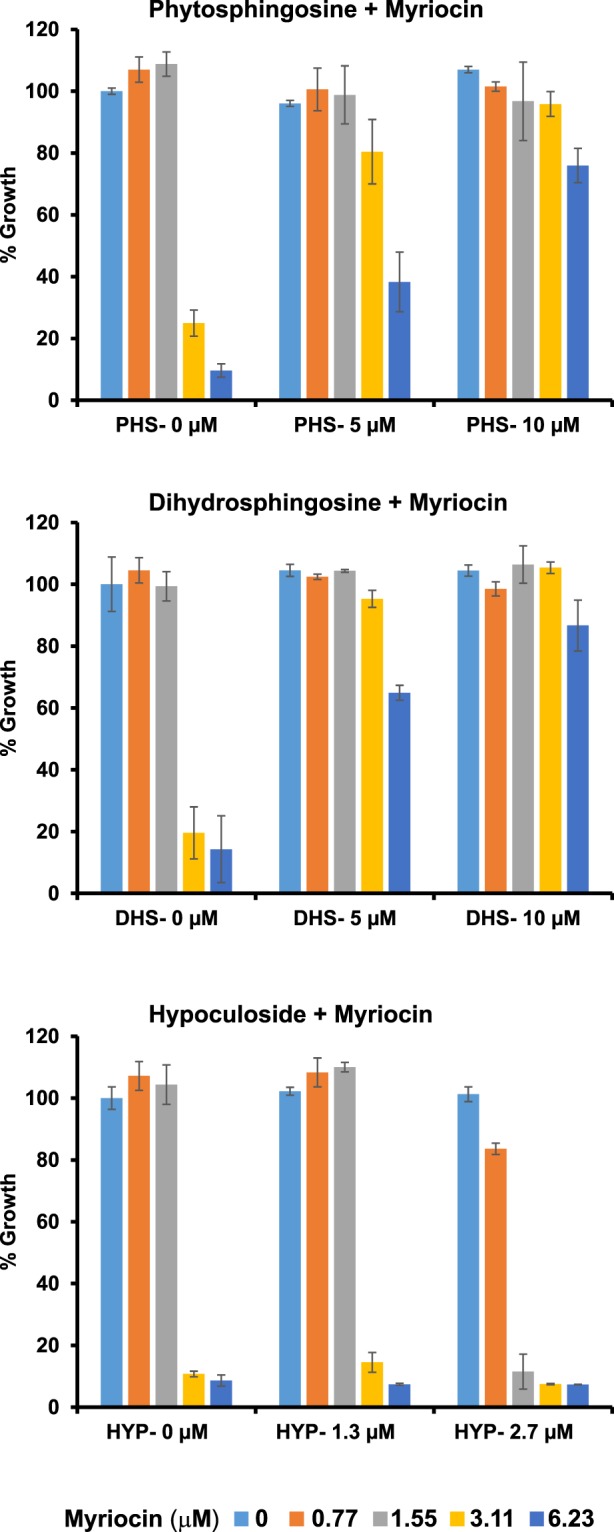
Hypoculoside does not rescue the growth inhibition caused by myriocin. Wild type strain (BY4743) was grown in YPD medium containing different concentrations of myriocin and either phytosphingosine (PHS) or dihydrosphingosine (DHS) or hypoculoside (HYP) at the indicated concentrations. Normalized growth at the different concentrations after 24 hours of incubation at 30 °C is plotted for the various cultures. Representative data from three independent experiments are shown in the Figure.

Myriocin is an inhibitor of Serine palmitoyltransferase (SPT), which catalyzes the first step in the synthesis of sphingolipid biosynthesis. Addition of PHS or DHS can rescue inhibition caused by myriocin^28^. We tested whether hypoculoside can rescue the toxicity of myriocin–treated cells. Myriocin inhibited the growth of yeast cells at 3.11 μM (Fig. 6). Addition of either PHS or DHS at 10 μM completely rescued the myriocin-induced inhibition (Fig. 6). In contrast, hypoculoside did not rescue the myriocin-induced inhibition but had an additive effect (i.e. total effect of hypoculoside and myriocin = sum of their individual effects) on growth (Fig. 6). These results indicate that hypoculoside cannot compensate for other sphingosines in yeast cells and it inhibits a pathway distinct from the one targeted by myriocin.

### Hypoculoside disrupts the vacuolar structure of yeast cells

**Figure 7:**
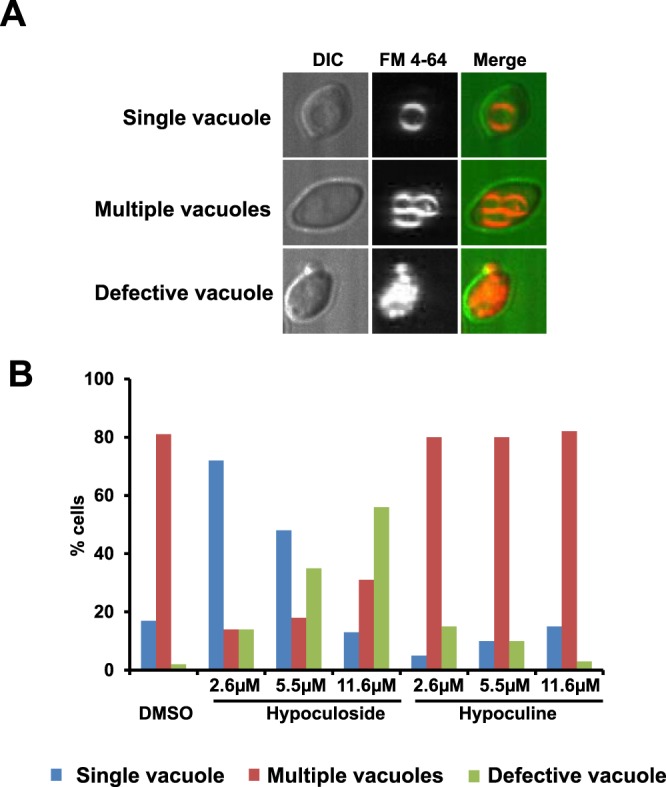
Hypoculoside but not hypoculine disrupts the vacuolar structure of yeast cells. Wild type yeast cells containing vacuoles pre-labelled with the lipophilic dye FM 4-64 were exposed to either DMSO or hypoculoside or hypoculine at the indicated concentrations for 1 hour. Effects of the compounds on vacuolar morphology was analyzed by fluorescence microscopy. (**A**) Based on the FM 4–64 staining patterns, yeast cells were classified into three categories: Single vacuole or multiple vacuoles or defective vacuoles. Representative images for each category are depicted. (**B**) Quantification of the various categories for cells treated with either DMSO or hypoculoside or hypoculine is plotted (N = 100 cells). Data from an independent experiment are presented in Supplementary Fig. S13.

As several vacuolar transport mutants were sensitive to hypoculoside (Fig. 3), we examined the effect of hypoculoside on the vacuolar structure of yeast cells. We incubated wild type yeast cells with FM 4–64, a dye that stains the vacuolar membranes^29^, for 1 hour. We then washed off the excess FM 4–64 from the medium and transferred the cells into fresh medium with either hypoculoside or hypoculine or DMSO. We classified the cells into three categories namely those that had a single vacuole or those with multiple vacuoles or those with defective vacuoles (Fig. 7A). About 80% of DMSO-treated cells had multiple vacuoles and the remaining 20% cells had a single vacuole (Fig. 7B). In contrast, about 50% of 11.6 µM hypoculoside-treated cells had defective vacuoles (Fig. 7B). However, only 3% of 11.6 µM hypoculine-treated cells had defective vacuoles. Thus, the effect of hypoculoside and hypoculine on vacuolar structure correlates with their inhibitory activities.

### Hypoculoside affects plasma membrane integrity of yeast cells

**Figure 8:**
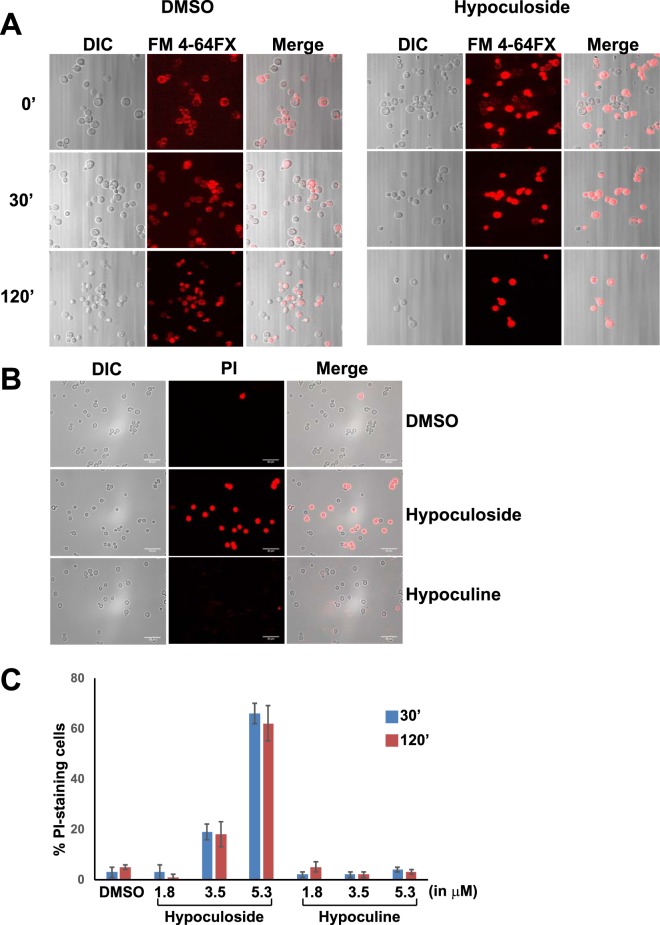
Hypoculoside but not hypoculine disrupts the membrane integrity of yeast cells. (**A**) Wild type yeast cells pre-treated with either DMSO or hypoculoside (5.4 μM) were exposed to FM 4-64FX. Uptake of FM 4-64FX after 0′, 30′ and 120′ was analyzed by fluorescence microscopy of fixed cells. (**B**) Yeast cells were incubated with either DMSO or hypoculoside or hypoculine at different concentrations (1.8, 3.5 and 5.3 µM) for 30′ and 2 hours. They were then treated with propidium iodide (PI) and percentage of PI-staining cells was assayed by fluorescence microscopy. Representative images of cells visualized by bright field and fluorescence microscopy are shown along with quantification of PI-staining cells (N = 100 cells) in (**C**). Representative data from three independent experiments are shown in the Figure.

To test if hypoculoside affects endocytosis, we used a dye FM 4–64FX which is a fixable analog of the FM 4-64 membrane stain. We incubated logarithmically growing yeast cells with either DMSO or hypoculoside for 30 minutes and then washed the cells off the compound and resuspended them in fresh growth medium containing FM 4-64FX to visualize endocytosis and vacuolar structure. In DMSO pre-treated cells, FM 4-64FX first stained the plasma membrane (Fig. 8A) at t = 0 and stained the cytoplasmic structures after 30′ indicating its uptake by endocytosis (Fig. 8A). However, in hypoculoside pre-treated cells, FM 4-64FX stained the cytoplasmic contents even at t = 0 (Fig. 8A). These results indicate that hypoculoside compromised the membrane integrity of yeast cells.

### Hypoculoside has membrane disrupting activity

We then tested whether hypoculoside causes membrane leakage using the membrane impermeant dye propidium iodide (PI). Yeast cells were treated with either hypoculoside or its non-toxic aglycone derivative hypoculine at different concentrations (1.8, 3.5 and 5.3 µM) or with DMSO, for 30′ and 2 hours. We then incubated the cells with PI and visualized its uptake by fluorescence microscopy. 98% of DMSO-treated cells failed to take up PI as expected (Fig. 8B,C). However, about 60% of cells treated with hypoculoside (5.3 µM) had internalized PI after 2 hours (Fig. 8B,C). In contrast, less than 10% of aglycone–treated cells had PI staining (Fig. 8B,C). These results indicate that hypoculoside disrupts the membrane integrity of yeast cells.

## Discussion

We report the discovery of a new deoxysphingoid base-related compound from *Acremonium* with antifungal, antimicrobial and cytotoxic activities. To the best of our knowledge, such a deoxysphingoid base has never been reported from any organism. Chemogenomic profiling analysis of hypoculoside in budding yeast indicated that it is two-fold more toxic to several vesicular trafficking mutants in comparison to the wild type strain. Interestingly, the sugar residue in hypoculoside is essential for its toxicity. This is in stark contrast to oceanipiside, another deoxysphingoid derivative whose aglycone derivative is more toxic^16^.

Multiple lines of evidence suggest that hypoculoside disrupts the membranes of yeast cells. Firstly, hypoculoside has a cytocidal activity on yeast cells. Secondly, hypoculoside renders yeast cells permeable to propidium iodide which normally cannot cross the yeast plasma membrane. Thirdly, pre-treatment of yeast cells with hypoculoside resulted in immediate staining of intracellular membranes by the lipophilic dye FM 4-64. This is in contrast to control cells in which intracellular staining is only detected after 30 minutes as internalization of the dye requires endocytosis. Fourthly, hypoculoside disrupts the vacuolar structure of yeast cells. Vacuoles in yeast disintegrate in response to osmotic stress, which is thought to help in their adaptation^30^. Our observations are consistent with the idea that hypoculoside causes osmotic stress by causing membrane damage. Moreover, a number of vacuolar transport mutants were sensitive to the inhibitory action of hypoculoside.

Although we have focused on the antifungal activity of hypoculoside, it also displays antibacterial and cytotoxic activities. Hypoculoside inhibited the growth of the Gram-positive bacterium *Staphyloccus aureus* but not the Gram-negative bacteria *Pseudomonas aeruginosa* and *Klebsiella aerogenes*. It would be interesting to test whether hypoculoside also inhibits bacteria and mammalian cells by perturbing their plasma membranes. Interestingly, sphingosine, dihydrosphingosine and phytosphingosine have been shown to inhibit the growth of *Staphylococcus aureus* and disrupt cell membrane^31^.

It would be interesting to compare how hypoculoside and hypoculine interact with the plasma membrane and assess their relative effects on membrane fluidity and structure. The sugar group in hypoculoside might make it more amphipathic in comparison to hypoculine and facilitate its insertion into the lipid bilayer. It is equally intriguing to determine the biological function(s) of hypoculoside in *Acremonium* sp. F2434. It could either modulate the membrane structure or act as a signaling molecule or confer protection from other microbes.

Determining the biosynthetic pathway of hypoculoside in *Acremonium* sp. F2434 would be informative. Based on the biosynthetic pathways of related molecules like sphingosine and fumonisin B1, we hypothesize that hypoculoside biosynthesis involves addition of alanine to a hydrocarbon chain. This would explain the presence of -NH_2_ and -CH_3_ groups attached to the C23 in hypoculoside (compound 8: Fig. 2B). Two enzymes Fum8 and Serine palmitoyltransferase (SPT) that belong to the family of 2-oxoamine synthases have been shown to catalyze the addition of alanine to a hydrocarbon chain. Fum8, an aminotransferase involved in fumonisin B1 biosynthesis in the fungal maize pathogen *Fusarium verticillioides*, adds alanine onto the polyketide chain^32,33^. SPT catalyzes the first step in sphingosine biosynthesis namely the condensation of serine with palmitoyl CoA^34^. Although serine is the preferred substrate of SPT, alanine can be utilized at a low frequency and mutations in SPT have been shown to promote the use of alanine instead of serine in the condensation reaction^35^. It is therefore tempting to speculate that a homologue of Fum8/SPT in *Acremonium* might be involved in hypoculoside biosynthesis. The hydrocarbon chain itself could be synthesized either by a fatty acid synthase or a Polyketide synthase (PKS). Uncovering genes involved in hypoculoside biosynthesis in *Acremonium* and testing the phenotypic consequences of their inactivation are exciting but formidable challenges for the future.

## Methods

### Fermentation, extraction and isolation of hypoculoside

The fungal strain F2434 was isolated from a soil sample collected in Singapore. F2434 was sub-cultured on Malt Extract Agar (Oxoid, CM0059) for 7 days at 24°C. Three agar plugs of 5 mm diameter from the culture plate was then used to inoculate each of 40 × 250 mL Erlenmeyer flasks, each containing 50 mL of fermentation medium [sucrose 30 g/L (Merck), soluble starch 10 g/L (Sigma), malt extract 5 g/L (Sigma), yeast extract 5 g/L (Becton Dickinson), calcium carbonate 5 g/L (Sigma), vegetable juice 200 mL/L (Campbell V8), pH 6.5]. These cultures were allowed to grow for 14 days at 24  °C with shaking at 200 rpm. At the end of the incubation period, cultures from all 40 flasks were harvested and freeze dried. The freeze-dried cultures were then extracted overnight with an equal volume of 1:1 dichloromethane: methanol. The entire extraction mixture was then passed through cellulose filter paper (Whatman Grade 4) to remove the solid materials, and the filtrate was subsequently dried using a rotary evaporator.

The dried crude extract (9 g) was then re-dissolved in 8 mL of methanol and separated by reverse-phase preparative HPLC column with the gradient elution program (15% B isocratic for 5 minutes; 15% to 20% B over 5 minutes, followed by 20% to 40% B over 50 minutes, and an increase from 40% to 60% B over 20 minutes, and to 100% B in 10 minutes) generating forty fractions. Bioactivity-guided analysis of various fractions was performed by testing the effect of individual fraction on the growth of *Candida albicans* (ATCC 900280) as previously described^18^. Antifungal activity against *Candida albicans* was found to be concentrated in fraction eluting at 46–46.5 min. This fraction was concentrated and dried under reduced pressure to give hypoculoside (16 mg) as a white amorphous solid.

### Purification and chemical characterization of hypoculoside

Preparative HPLC analysis was performed on the Agilent 1260 Infinity Preparative-Scale LC/MS Purification System, completed with Agilent 6130B single quadrupole mass spectrometer for LC and LC/MS Systems. The samples were separated on an Agilent Prep C18 column (100×30 mm) at flowrate of 30 mL/min by gradient elution with a mixture of 0.1% formic acid in water (solvent A) and 0.1% formic acid in acetonitrile (solvent B). The HRESIMS and MS/MS spectra were acquired on Agilent UHPLC 1290 Infinity coupled to Agilent 6540 accurate-mass quadrupole time-of-flight (QTOF) mass spectrometer equipped with a splitter and an ESI source. The analysis was performed with a C18 4.6 × 75 mm, 2.7 µm column at a flowrate of 2 mL/min, under standard gradient condition of 5% to 100% acetonitrile with 0.1% formic acid over 14 minutes. The typical QTOF operating parameters were as follows: positive ionization mode; sheath gas nitrogen, 12 L/min at 295 °C; drying gas nitrogen flow, 8 L/min at 275 °C; nebulizer pressure, 30 psig; nozzle voltage, 1.5 kV; capillary voltage, 4 kV. Lock masses in positive ion mode: purine ion at *m/z* 121.0509 and HP-921 ion at *m/z* 922.0098. MS/MS data of precursor ions were acquired using collision energies between 10–40 eV and acquisition rate at 2 spectra/s. MS/MS analysis was performed with a Zorbax Eclipse Plus 2.1 × 50 mm 1.8 µm column at flowrate of 0.5 mL/min, under standard gradient condition of 5% to 100% acetonitrile with 0.1% formic acid over 10 minutes. NMR spectra were collected on a Bruker DRX-400 NMR spectrometer with Cryoprobe, using 5-mm BBI (^1^H, G-COSY, multiplicity-edited G-HSQC, and G-HMBC spectra) or BBO (^13^C spectra) probe heads equipped with z-gradients. Spectra were calibrated to residual protonated solvent signals (CD_3_OD δ_H_ 3.30 and CD_3_OD δ_C_ 49.0). Optical rotations were recorded on a JASCO P-2000 digital polarimeter. UV spectra were obtained on a GE Healthcare Ultrospec 9000 spectrophotometer.

### Hydrolysis of hypoculoside

Acid-hydrolysis was performed as previously described with minor modifications^36^. Hypoculoside (**8**, 10 mg) was hydrolyzed in 4 M HCl (2 mL) and refluxed for 5 hours. Then the reaction flask was cooled on ice water for 3 min and 2 mL of water was added to the flask. The reaction mixture was then neutralized with ammonia. The sugar was separated from the aglycone by C18 Reversed-phase column chromatography (Phenomenex, Sepra C18-E, 50 µm, 65 A). The sugar was first eluted by using 20 mL water. The aglycone was subsequently eluted by addition of 40 mL methanol. The identity of the sugar was determined by comparing its retention time (peak detected at 20 min) with that of the authentic standard sugars by HPLC on Shodex KS-G and KS-801 columns with 100% water as mobile phase. A perfect match with the retention time of D-glucose established that hypoculoside contains D-glucose. The aglycone obtained from the 40 mL methanol eluted fraction was dried under reduced pressure to give a white amorphous solid (7 mg) which we named as hypoculine (**9**).

### Chemical structural data

The NMR spectra of hypoculoside and hypoculine are provided in Supplementary Figs S3–S12.

**Hypoculoside (8)** white amorphous solid; [α]_D_ + 40.5 (c 1.2, MeOH); UV (CH_3_CN) λ_max_ (log ε) end absorption nm; HRESIMS m/z at 564.4474 [M + H]+ (calcd for C_30_H_61_NO_8_+H, 564.4475); HRESIMS/MS m/z 402.3943 [M-C_6_H_10_O_5_+H]+, m/z 384.3844 [M-C_6_H_10_O_6_-H_2_O+H]+, m/z 366.3730 [M-C_6_H_10_O_6_-2H_2_O+H]+, m/z 348.3629 [M-C_6_H_10_O_6_-3H_2_O+H]+; ^1^H and ^13^C NMR data, see Table 1.

**Hypoculine (9)** white amorphous solid; [α]_D_ + 67.0 (c 0.5, MeOH); UV (CH_3_CN) λ_max_ (log ε) end absorption nm; HRESIMS m/z at 402.3950 [M + H]^+^ (calcd for C_24_H_51_NO_3_+H, 402.3947); ^1^H and ^13^C NMR data, see Table 1.

### rRNA and ITS1 sequencing of the hypoculoside-producing fungus

To clarify the phylogenetic status of the hypoculoside-producing fungus F2434, we PCR amplified and sequenced its 18 S rRNA and ITS1 regions. Genomic DNA was isolated form hypoculoside-producing fungus F2434 using MagListo™ 5 M Plant Genomic DNA Extraction Kit (Bioneer). ITS2 was amplified using KAPA HiFi HotStart ready mix (KAPA biosystems) with 0.4 µM of primers, ITS86F 5′GTGAATCATCGAATCTTTGAA3′ and ITS4 5′ TCCTCCGCTTATTGATATGC3′^37^. PCR conditions were as follows: initial denaturation at 95 °C for 180 s, followed by 34 cycles of 98 °C 20 s, 52 °C 30 s and 72 °C 60 s and a final extension phase at 72 °C for 600 s. 18 S rRNA was amplified using KAPA HiFi HotStart ready mix (KAPA biosystems) with 0.3 µM of primers, 574 F 5′CGGTAAYTCCAGCTCYV3′ and 1132 R 5′CCGTCAATTHCTTYAART3′^38^. PCR conditions were as follows: initial denaturation at 95 °C for 180 s, followed by 34 cycles of 98 °C 20 s, 52 °C 30 s and 72 °C 60 s and a final extension phase at 72 °C for 600 s. Sequences of F2434 18 S rRNA and ITS1 were deposited at GENBANK with the accession numbers MH179060 and MH179059 respectively. Phylogenetic analyses of the internal transcribed spacer (ITS) gene sequences was carried out by applying the maximum-likelihood algorithm^39^ using Mega X^40^ and bootstrap values based on 500 replications^41^ and the results showed that F2434 was a novel species belonging to the genus *Acremonium* (Supplementary Fig. S14).

### Inhibition of growth assays in *C*. *albicans* and *S*. *cerevisiae*

Susceptibility of *Candida albicans* strain SC5314 to hypoculoside and amphotericin B was determined by a broth microdilution assay using Clinical Laboratory Standards Institute (CLSI) guidelines^42^. Cells grown to exponential phase in YPD medium (1% Yeast extract, 2% Bacto peptone and 2% glucose) were harvested, washed and resuspended in RPMI 1640 medium at a density of 2×10^3^ cells/mL. RPMI 1640 medium with L-glutamine without sodium bicarbonate (Sigma) was buffered with 0.165 M morpholinepropanesulfonic acid (MOPS) to a pH of 7.0. Two-fold dilutions of hypoculoside and amphotericin B were prepared such that final concentration of DMSO was 1% in all cultures. Cell suspension was distributed in microtiter plate (200 µL/well) and incubated at 35 °C with 220 rpm for 24 h. The *Saccharomyces cerevisiae* diploid wild-type strain (BY4743) was used for determining the Inhibitory Concentration (IC) of hypoculoside. Frozen yeast cells were allowed to recover on YPD agar plates and grown in YPD medium for nine generations (OD_600 nm_ ≤ 2). Cells were diluted to OD_600 nm_ of 0.0625 in YPD medium. 200 μL of the diluted yeast culture was transferred into the 96-well microtiter plate having 2-fold serially diluted concentrations of hypoculoside. Cells were incubated in a microplate reader for 16–24 hours at 30 °C with shaking. IC of hypoculoside was computed by comparing the growth of treated versus control cells.

### Antibacterial and mammalian cell cytotoxicity assays

Effect of hypoculoside on the growth of three bacterial strains *Staphylococcus aureus* (ATCC 25923), *Pseudomonas aeruginosa* (ATCC 9027) and *Klebsiella aerogenes* (ATCC 13048) was assessed as previously described^43^. Cytotoxicity of hypoculoside on the A549 human lung cell line and the pancreatic carcinoma cell lines MIA PaCa-2 and PANC-1 was determined as described before^43^. A549 and MIA PaCa-2 were seeded at 1,500 cells per well, and PANC-1 cells were seeded at 2,500 cells per well in a 384-well microplate. Cells were treated with hypoculoside at various concentrations and incubated for 72 hours at 37 °C in the presence of 5% CO_2_. Cytotoxic effect of hypoculoside was measured using the PrestoBlue™ cell viability reagent (Life Technologies). Following incubation of the microplates with the dye for 2 hours, the fluorescence reading (Excitation/Emission: 560 nm/590 nm) was recorded using the Tecan Infinite M1000 Pro reader.

### Homozygous profiling (HOP) assay

HOP assay was done as described previously^44^ with a commercially available collection of pooled yeast homozygous Knock out collection (Invitrogen). For the assay, hypoculoside was used at 2.7 µM which caused a 60% reduction in growth in YPD medium after 10 hours at 30 °C. The uptag barcode was amplified from genomic DNA prepared from DMSO-treated and hypoculoside-treated yeast cells using a common reverse oligo R_c_ and a variable forward oligo F_v_ containing a sample-specific TruSeq index. Sequence of R_c_ is 5′AATGATACGGCGACCACCGAGATCTACACTCTTTCCCTACACGACGCTCTTCCGATCT GTCCACGAGGTCTCT 3′. Sequence of F_v_ is 5′ CAAGCAGAAGACGGCATACGAGATNNNNNN GTGACTGGAGTTCAGACGTGTGCTCTTCCGATCTGTCGACCTGCAGCGTACG 3′. Nucleotides indicated in green in R_c_ and F_v_ are complementary to sequences flanking the bar-code in the Yeast knockout strains. Nucleotides indicated in red in R_c_ is the sequence of the Universal Illumina Adaptor. Nucleotides in blue in F_v_ is the sequence of the Illumina PCR Primer 2.0. Hexanucleotide NNNNNN in F_v_ is the sample-specific TruSeq index. Nucleotides indicated in orange in F_v_ is sequence of the Multiplexing Read 2 sequencing primer. PCR products were pooled and sequenced on MiSeq System (Illumina) with 1 × 51 single-end reads. Sequencing data was analyzed as described previously^44^. NGS data was deposited at the NCBI’s Sequence Read Archive (SRA) database (accession: PRJNA498909).

### Bioinformatic methods

Gene Ontology analysis was performed using the online tools DAVID^25,26^ and REVIGO^27^.

### FM 4-64FX Fluorescence microscopy

Labelling of yeast cells with FM4-64FX (Invitrogen) was done as previously reported^45^. Overnight culture of yeast cells was diluted into fresh nutrient medium at OD_600 nm_ = 0.2 and grown for few hours until OD_600 nm_ = 0.8. Then, the cells were treated with either DMSO or 5.4 µM of hypoculoside for 30′, at 30 °C with shaking. Following that, the cells were spun down at 3000 × g, 5′ at 4 °C and washed once with 1 mL of cold YPD media. 20 μM of FM 4-64FX was then added to the cells and incubated on ice for 10′^46^. After that, the cells were washed with 1 mL of cold YPD medium and then incubated at 30 °C for 0′, 30′ and 120′ in a shaker, before fixation. The cells were fixed with 3.7% formaldehyde for 10 min on ice and washed with Phosphate Buffer Saline (PBS). The images were acquired using an inverted fluorescence microscope ZEISS LSM 5 LIVE (Carl Zeiss, Oberkochen, Germany).

### FM 4-64 Fluorescence microscopy

Labeling of yeast cells with FM4-64 (Invitrogen) was done as previously reported^47^. Overnight culture of yeast cells was diluted into fresh nutrient medium at OD_600 nm_ = 0.2 and grown for few hours until OD_600 nm_ = 0.8. Then, the cells were labeled with 15 µM FM4-64 in YPD for 1 hour, at 30 °C with shaking. After that, the cells were washed and treated with either DMSO or various concentrations of either hypoculoside or hypoculine for 30′, at 30 °C with shaking. Following that, the cells were spun down at 3000 × g, 5′ and washed once with 1 mL of Synthetic Defined (SD) media. After that, the cells were resuspended in 30 µL of SD media. The images were acquired using an inverted fluorescence microscope ZEISS LSM 5 LIVE (Carl Zeiss, Oberkochen, Germany).

### Propidium Iodide (PI)-staining assay

BY4743 cells (OD_600 nm_ ≈ 1) were exposed to various concentrations of hypoculoside or hypoculine or DMSO in YPD for 30′ and 2 hours. Cells were washed twice and resuspended in PBS and treated with 5 μg/mL PI for 20′ in the dark with shaking at 25 °C. PI uptake by cells was assayed by bright-field and fluorescence microscopy [Excitation/Emission (nm): 535/617] and the percentage of PI-staining cells (N ≥ 100) was calculated.

## Electronic supplementary material

Supplementary information

Table S1
