# Supplementary material for: Hypoculoside, a sphingoid base-like compound from Acremonium disrupts the membrane integrity of yeast cells
Source: Sci Rep. 2019 Jan 24;9:710. doi: 10.1038/s41598-018-35979-z (PMC6345779; doi:10.1038/s41598-018-35979-z)
Supplement: Supplementary file 1 — Supplementary information [file 41598_2018_35979_MOESM1_ESM.pdf]

Hypoculoside, a sphingoid base-like compound from *Acremonium* disrupts the membrane integrity of yeast cells

Mohammad Alfatah[1], Jin Huei Wong[1], Choy Eng Nge[1], Kiat Whye Kong[2], Kia Ngee Low[1], Chung Yan Leong[1], Sharon Crasta[1], Madhaiyan Munusamy[1], Amanda Mun Leng Chang[1], Shawn Hoon[2], Siew Bee Ng[1*], Yoganathan Kanagasundaram[1*] and Prakash Arumugam[1*]

1. Bioinformatics Institute, 30 Biopolis Street, #07-01, Matrix, Singapore 138671
2. Molecular Engineering Laboratory, 61 Biopolis Drive, #03-12 Proteos, Singapore 13867

*To whom correspondence must be addressed

Email: prakasha@bii.a-star.edu.sg , yoganathank@bii.a-star.edu.sg and ngsb@bii.a-star.edu.sg

Legends for Supplementary figures and tables

Figure S1 Analysis of hypoculoside by MS/MS

Collision-Induced dissociation (CID) MS/MS fragmentation spectra of hypoculoside (**8**) generated at energies of 10 eV, 20 eV and 40 eV are depicted in panels A, B and C respectively.

Figure S2 Hypoculoside has antifungal, antibacterial and mammalian cytotoxic activities.

A. Logarithmically growing *Candida albicans* cells were exposed to hypoculoside and amphotericin B at the indicated concentrations in triplicates in a 96-well microplate. A picture of the microplate after 24 hours of incubation at 30 °C is shown. Absorbance data for the experiment is displayed in Fig. 2C.
B. *Saccharomyces cerevisiae* cultures in duplicate were treated with hypoculoside at the indicated concentrations in YPD medium at 30 °C for 24 hours. Growth of the yeast cultures as measured by $OD_{600\ nm}$ is plotted against hypoculoside concentration. Yeast cells treated with either DMSO or 21 µM hypoculoside for 24 hours were then washed in YPD and plated at different dilutions on a YPD agar and incubated at 30 °C. The YPD agar plate was photographed after 2 days.
C. Growth inhibition data of hypoculoside against three bacterial strains *Staphylococcus aureus* (ATCC 25923), *Pseudomonas aeruginosa* (ATCC 9027) and *Klebsiella aerogenes* (ATCC 13048) are presented. Percentage growth is plotted against log (concentration of hypoculoside in µM).

D. Cytotoxicity data of hypoculoside against the A549, MIA PaCa-2 and PANC-1 mammalian cell lines obtained using the PrestoBlue™ cell viability reagent are presented. Percentage growth is plotted against log (concentration of hypoculoside in µM).

Figure S3 ^{1}H NMR spectrum (methanol-*d*$_4$, 400 MHz) of hypoculoside (**8**)

Figure S4 ^{13}C NMR spectrum (methanol-*d*$_4$, 100 MHz) of hypoculoside (**8**)

Figure S5 HSQC spectrum (methanol-*d*$_4$, 400 MHz) of hypoculoside (**8**)

Figure S6 COSY spectrum (methanol-*d*$_4$, 400 MHz) of hypoculoside (**8**)

Figure S7 HMBC spectrum (methanol-*d*$_4$, 400 MHz) of hypoculoside (**8**)

Figure S8 ^{1}H NMR spectrum (methanol-*d*$_4$, 400 MHz) of hypoculine (**9**)

Figure S9 ^{13}C NMR spectrum (methanol-*d*$_4$, 100 MHz) of hypoculine (**9**)

Figure S10 HSQC spectrum (methanol-*d*$_4$, 400 MHz) of hypoculine (**9**)

Figure S11 COSY spectrum (methanol-*d*$_4$, 400 MHz) of hypoculine (**9**)

Figure S12 HMBC spectrum (methanol-*d*$_4$, 400 MHz) of hypoculine (**9**)

Figure S13 Hypoculoside but not hypoculine disrupts the vacuolar structure of yeast cells Data from an independent experiment performed as described in Fig. 7 are presented here.

Figure S14 Phylogenetic analysis of the hypoculoside-producing fungus F2434 Phylogenetic Maximum Likelihood tree based on internal transcribed spacer (ITS) gene

sequences of F2434 fungus and members of the family *Plectosphaerellaceae* (consists of 11 genera). Species of the genera *Apoharknessia and Phaeoacremonium* were used as the out-group. Bootstrap values (expressed as percentages of 500 replications) greater than 20 % are shown at the branch points. Bar indicates 0.05 substitutions per nucleotide position.

Supplementary Table S1 HOP data of hypoculoside and GO analysis

Excel Worksheet ‘HOP_ Hypoculoside’ contains the raw HOP data indicating the logFC values for 3702 deletion strains along with the corresponding P-value, logCPM (Counts per Million), Likelihood Ratio (LR) and False Discovery Rate (FDR).

Excel Worksheet ‘P-Value <0.05, logFC<-0.5’ contains the list of 336 genes that confer resistance to hypoculoside.

Excel Worksheet ‘GO’ (Gene Ontology) contains enrichment analysis results for 336 genes obtained using DAVID for the GO categories namely, Biological Process (BP), Cellular Component (CC) and Molecular Function (MF).

Excel Worksheet ‘REVIGO’ contain the REVIGO Tree analysis of the overrepresented GO terms obtained with DAVID (GO terms with a P-value<0.05).

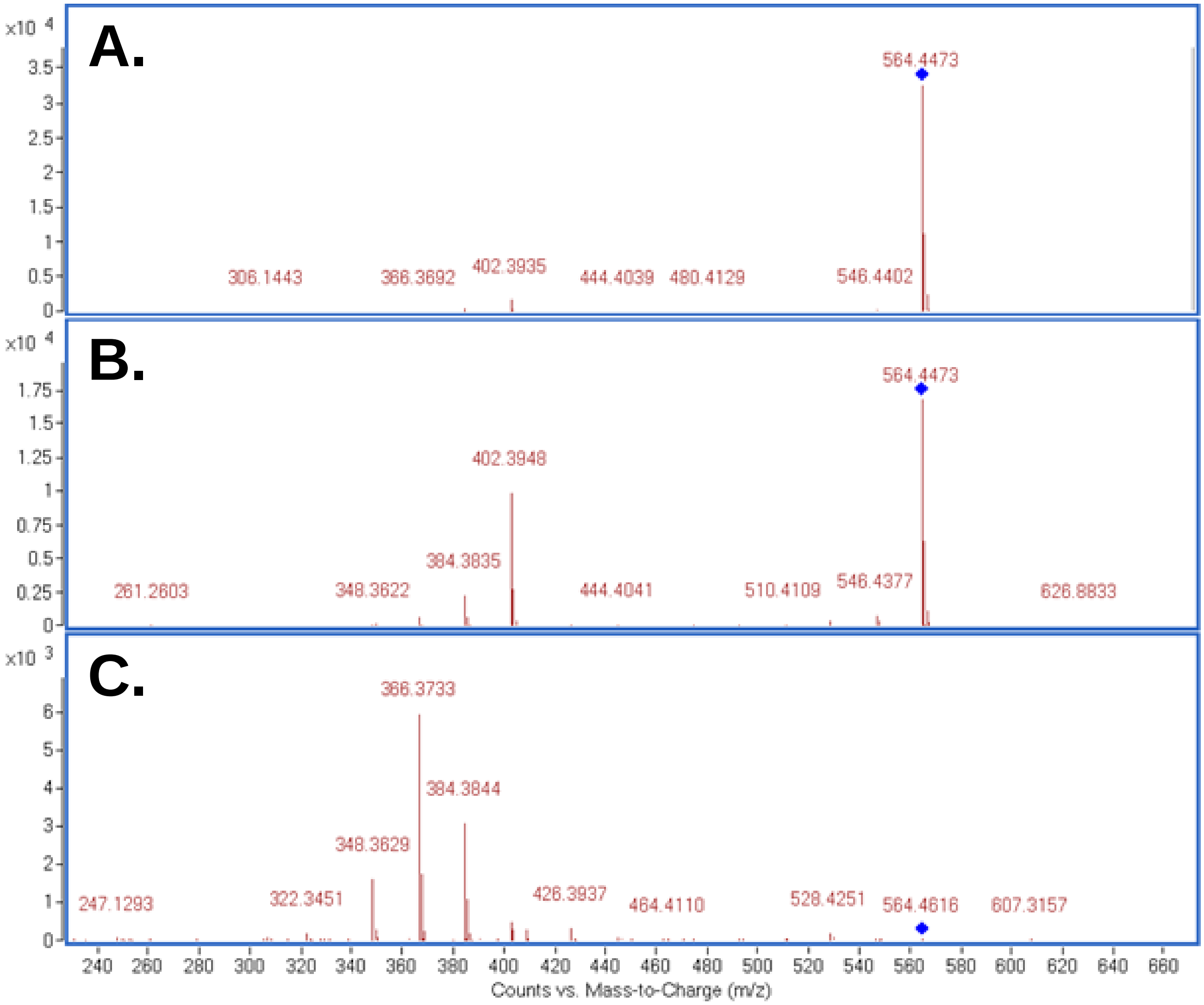

A.
x10 4
564.4473
306.1443
366.3692
402.3935
444.4039
480.4129
546.4402
B.
x10 4
564.4473
402.3948
384.3835
261.2603
348.3622
444.4041
510.4109
546.4377
626.8833
C.
x10 3
366.3733
384.3844
348.3629
247.1293
322.3451
426.3937
464.4110
528.4251
564.4616
607.3157
Counts vs. Mass-to-Charge (m/z)

Figure S1

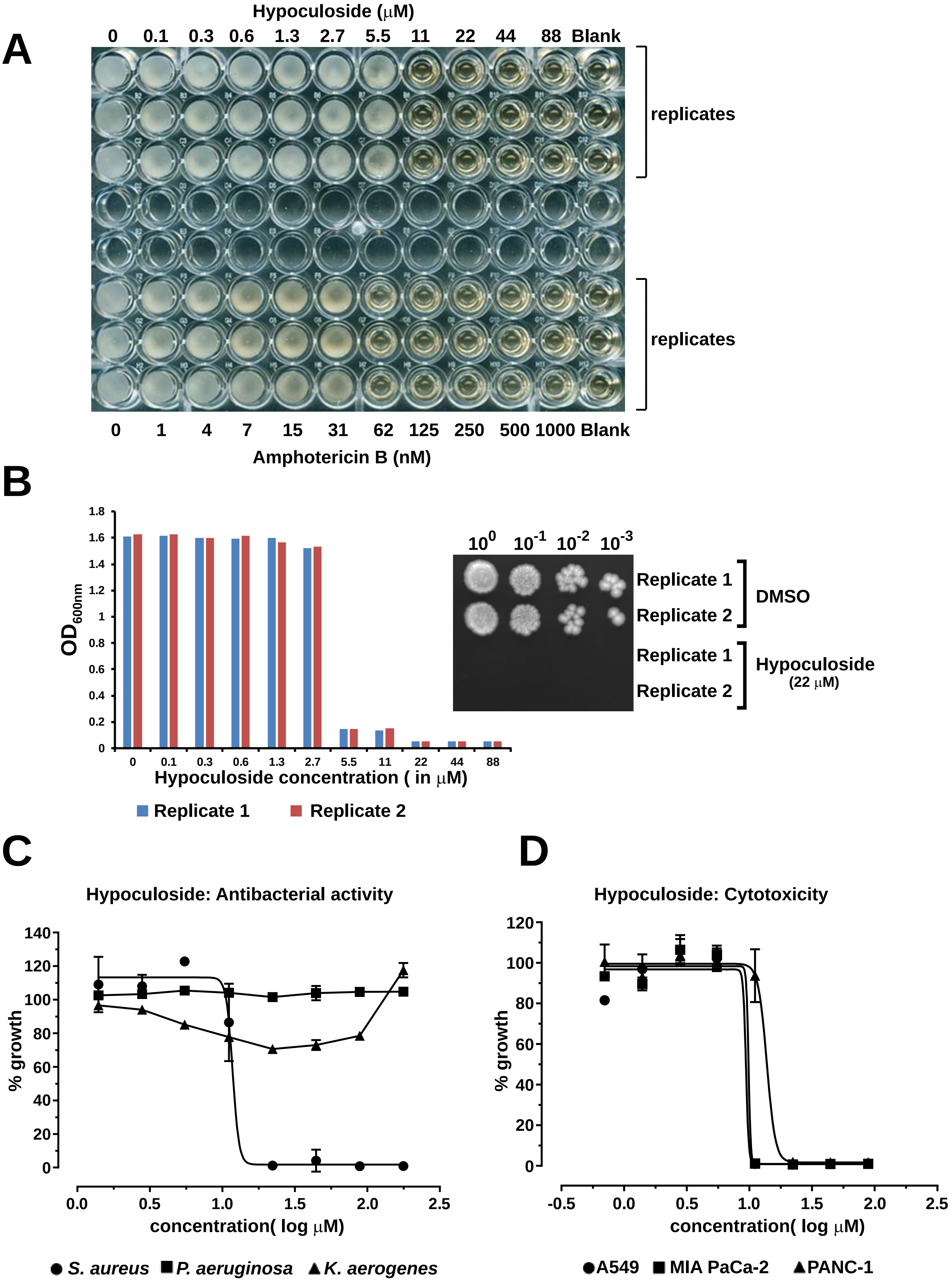
A
Hypoculoside (μM)
0 0.1 0.3 0.6 1.3 2.7 5.5 11 22 44 88 Blank
replicates
replicates
0 1 4 7 15 31 62 125 250 500 1000 Blank
Amphotericin B (nM)
B
OD600nm
Hypoculoside concentration (in μM)
Replicate 1
Replicate 2
10^0 10^-1 10^-2 10^-3
Replicate 1
Replicate 2
DMSO
Replicate 1
Replicate 2
Hypoculoside
(22 μM)
C
Hypoculoside: Antibacterial activity
% growth
concentration(log μM)
S. aureus
P. aeruginosa
K. aerogenes
D
Hypoculoside: Cytotoxicity
% growth
concentration(log μM)
A549
MIA PaCa-2
PANC-1

Figure S2

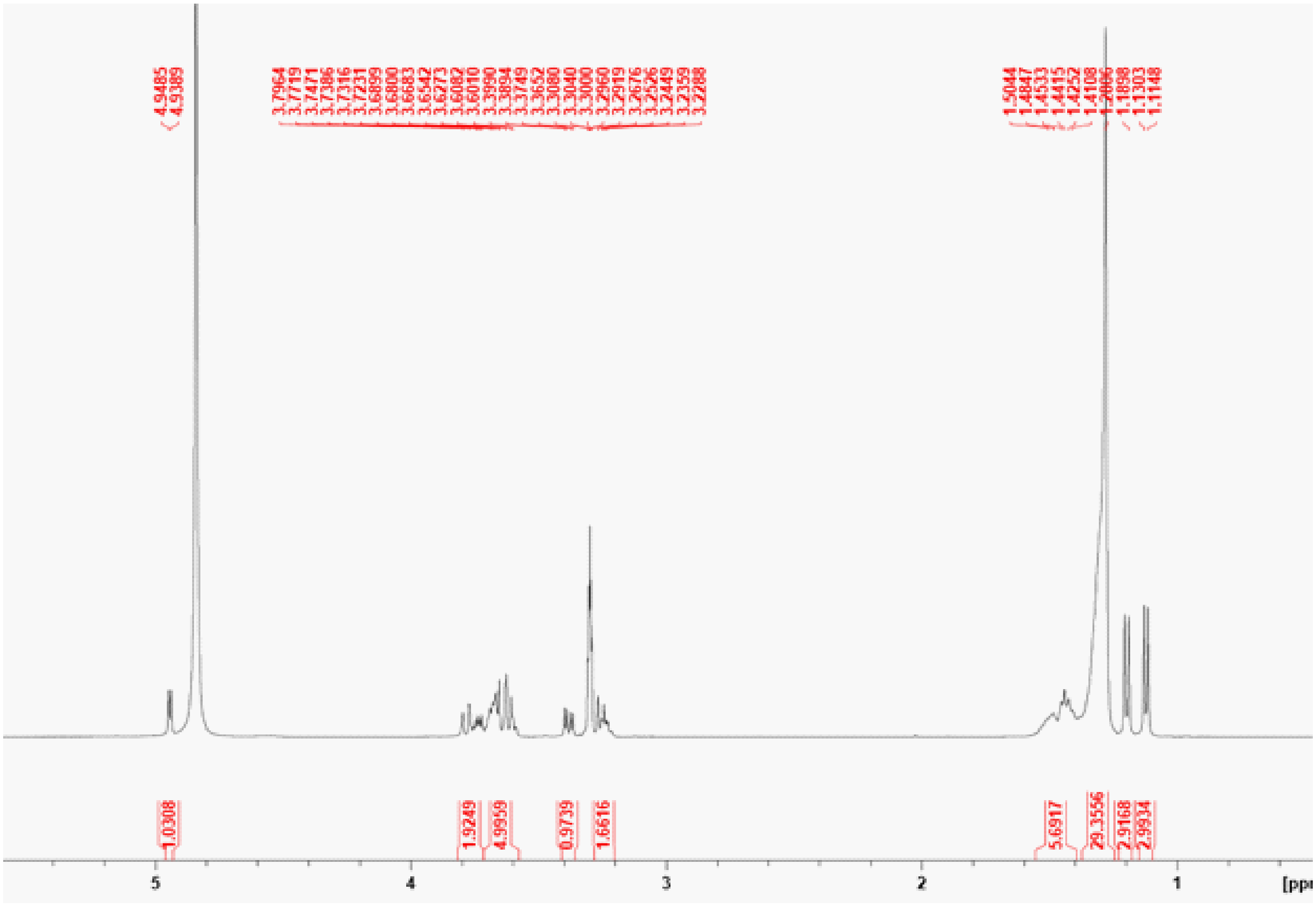
4.9485
4.9389
3.7964
3.7719
3.7471
3.7386
3.7316
3.7231
3.6899
3.6800
3.6683
3.6542
3.6273
3.6082
3.6010
3.3990
3.3894
3.3749
3.3652
3.3080
3.3040
3.3000
3.2960
3.2919
3.2676
3.2526
3.2449
3.2359
3.2288
1.5044
1.4847
1.4533
1.4415
1.4252
1.4108
1.1898
1.1303
1.1148
1.0308
1.9249
4.9959
0.9739
1.6616
5.6917
29.3556
2.9168
2.9934
5
4
3
2
1

Figure S3

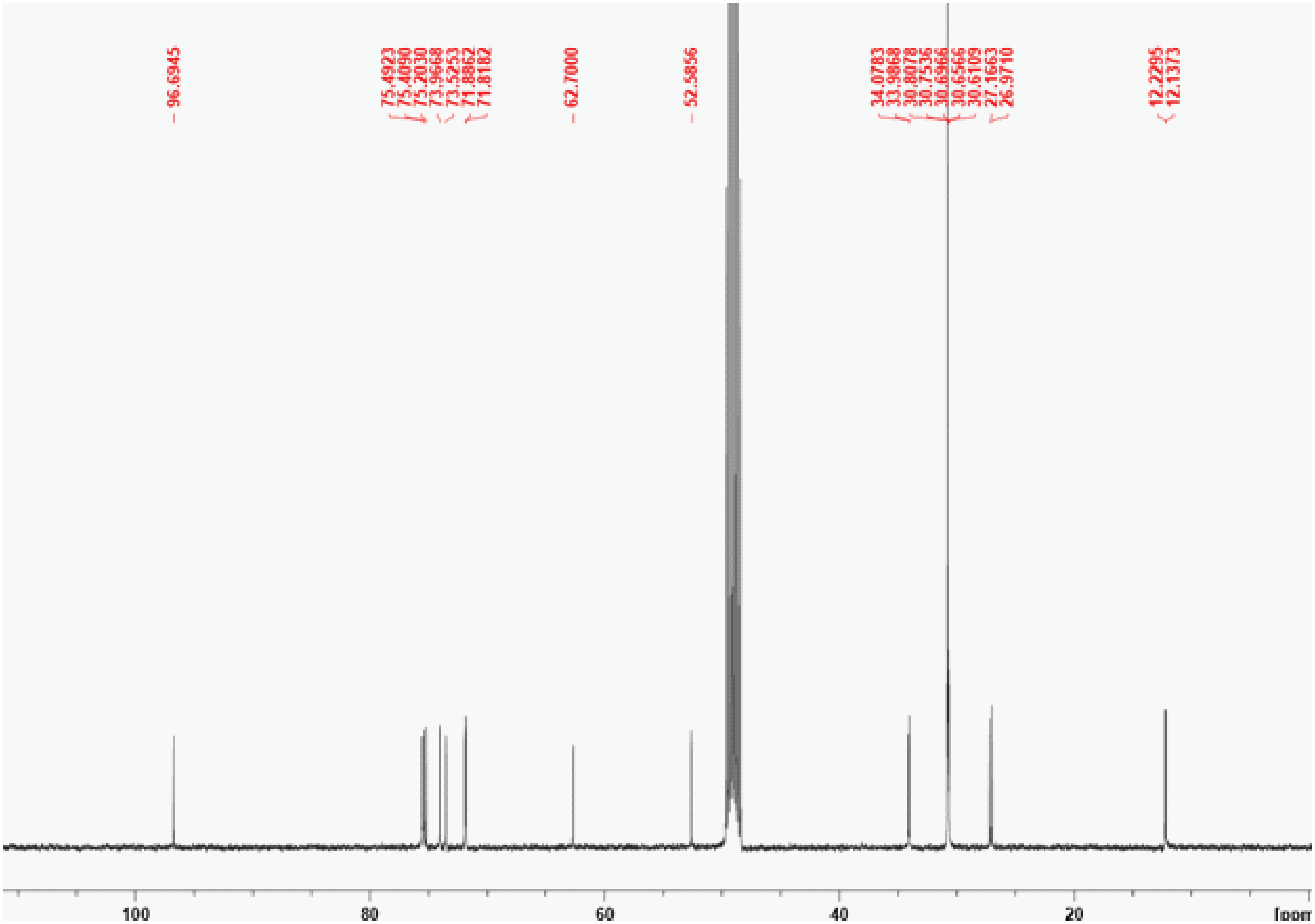

96.6945
75.4923
75.4090
75.2030
73.9668
73.5253
71.8862
71.8182
62.7000
52.5856
34.0783
33.9868
30.8078
30.7536
30.6966
30.6566
30.6109
27.1663
26.9710
12.2295
12.1373
100
80
60
40
20

Figure S4

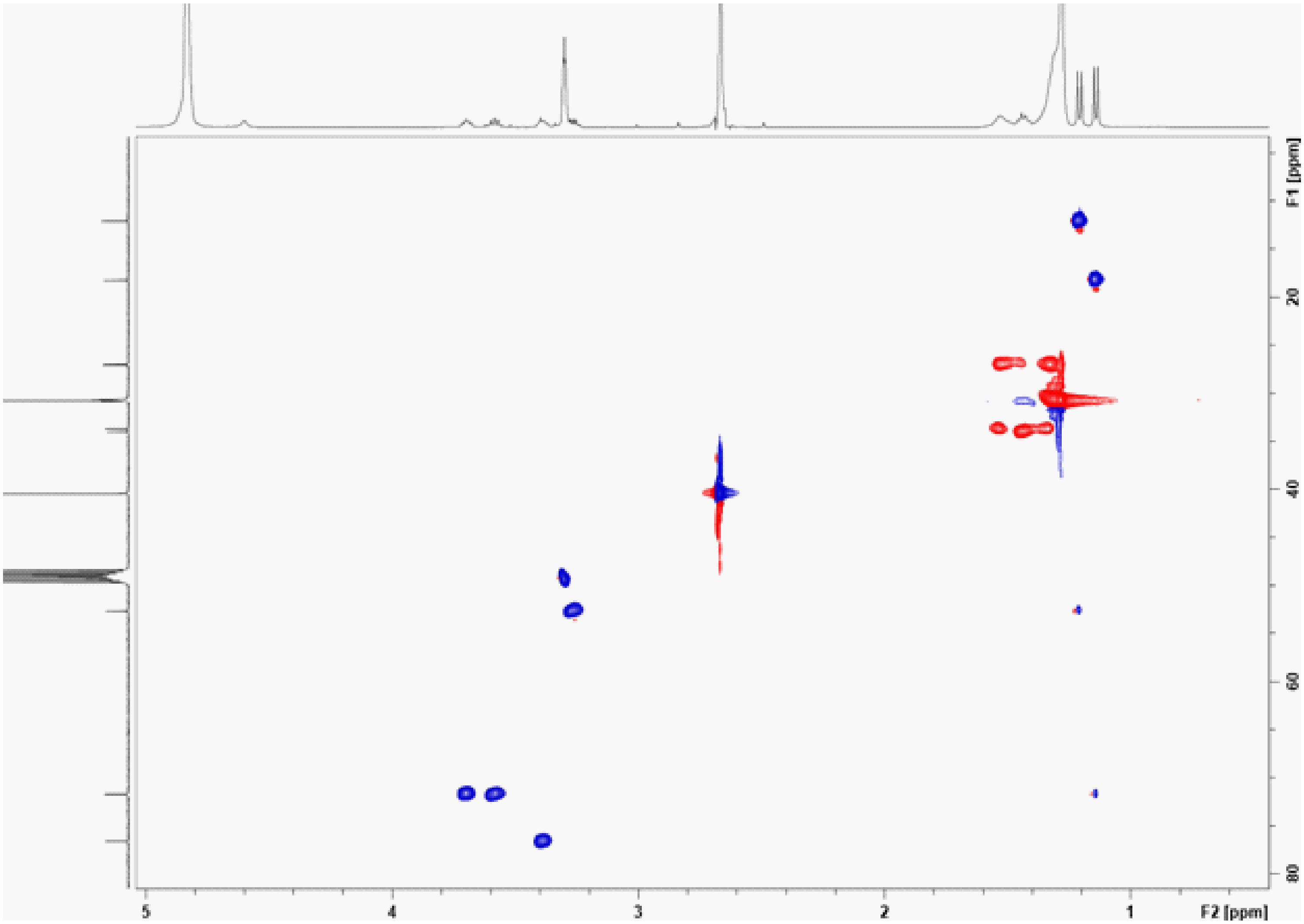

Figure S5

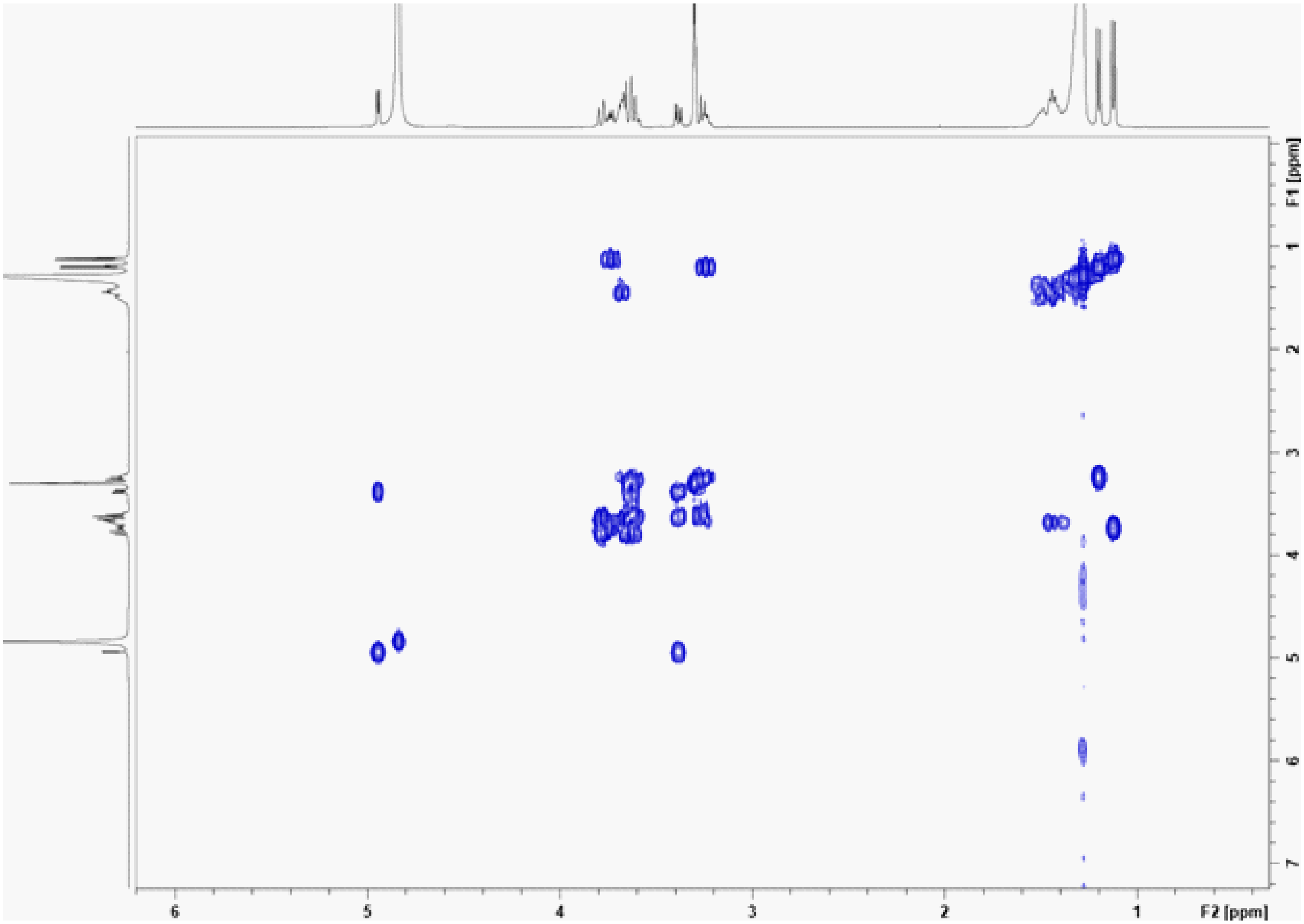

Figure S6

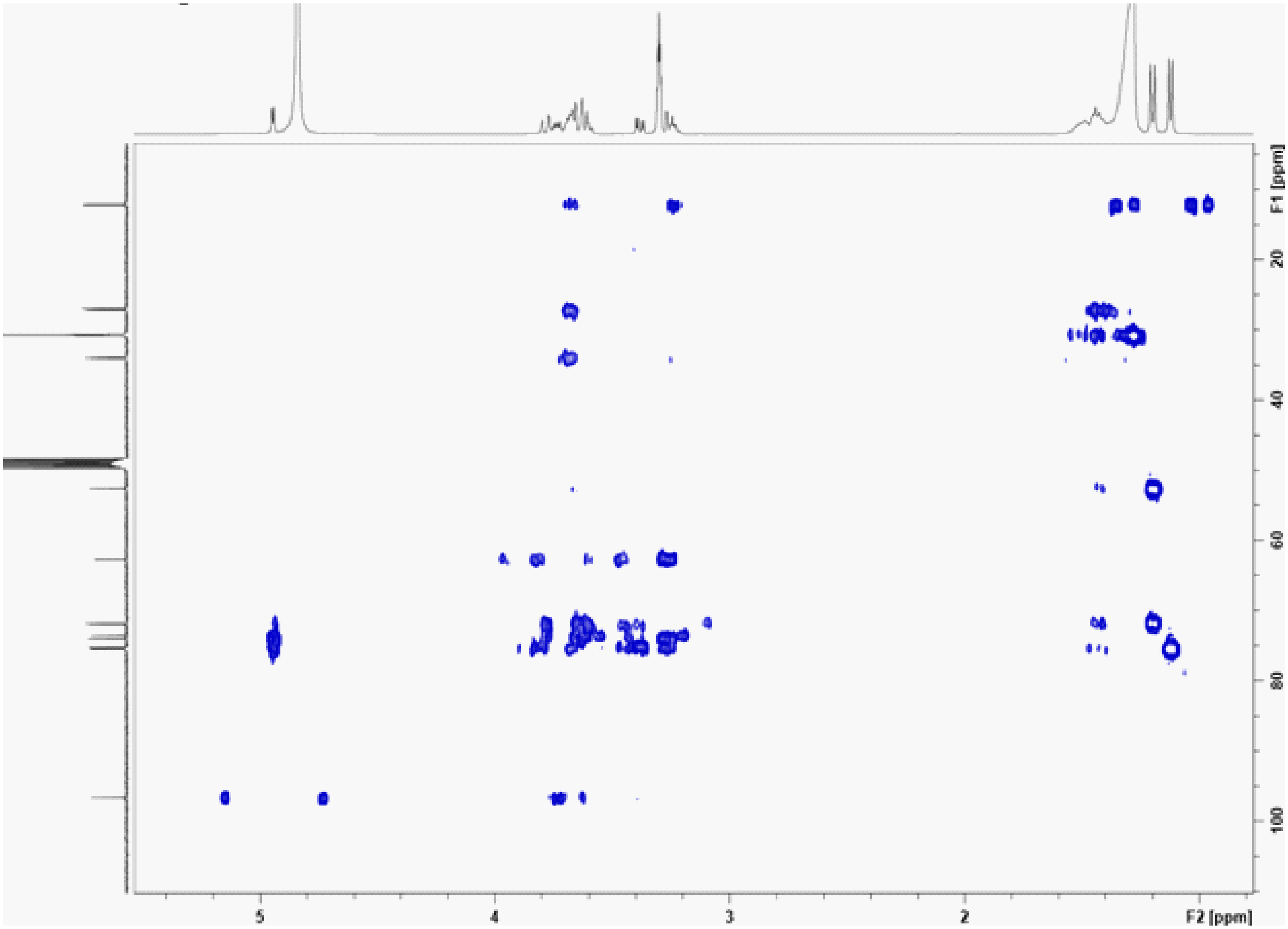

Figure S7

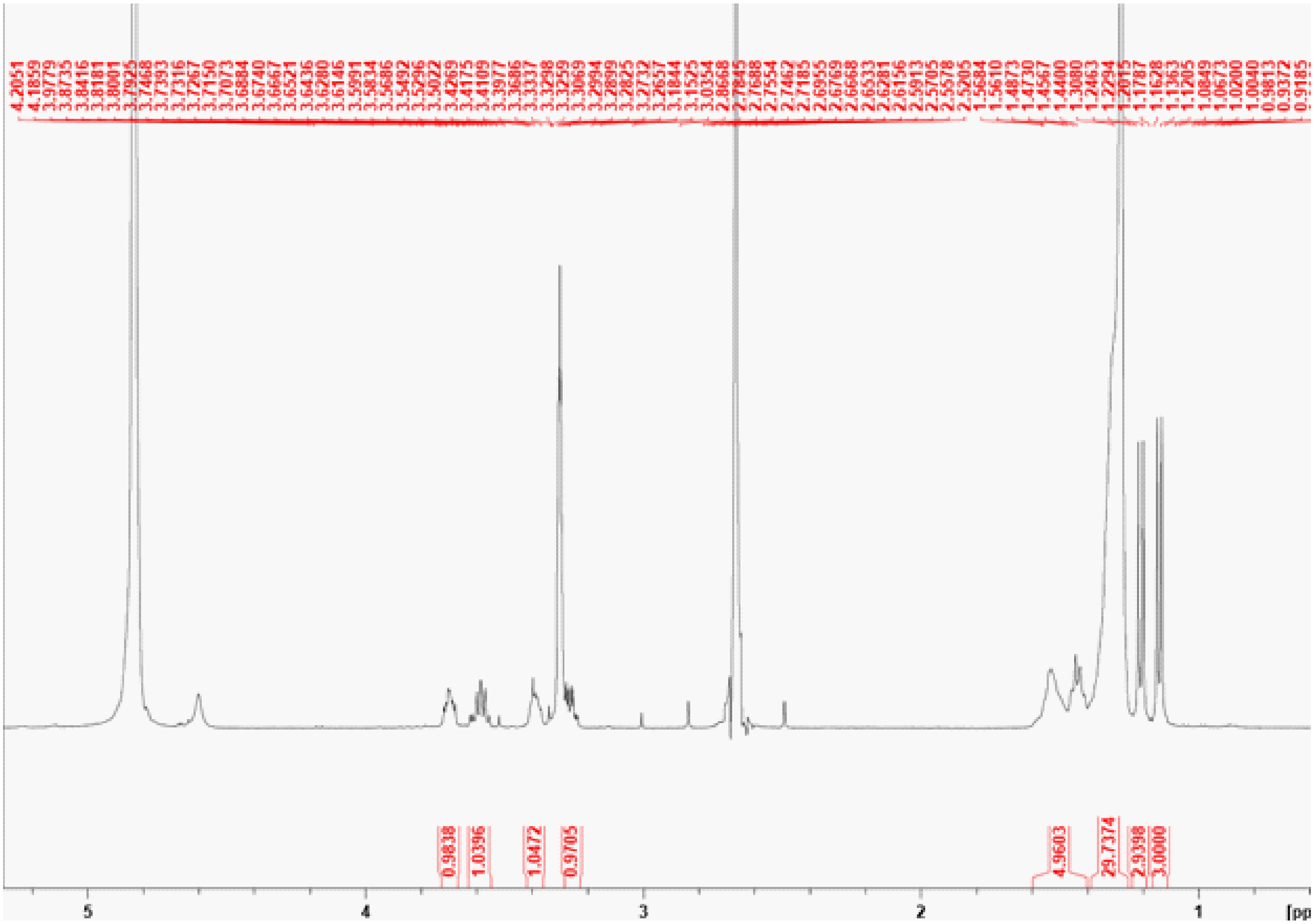

Figure S8

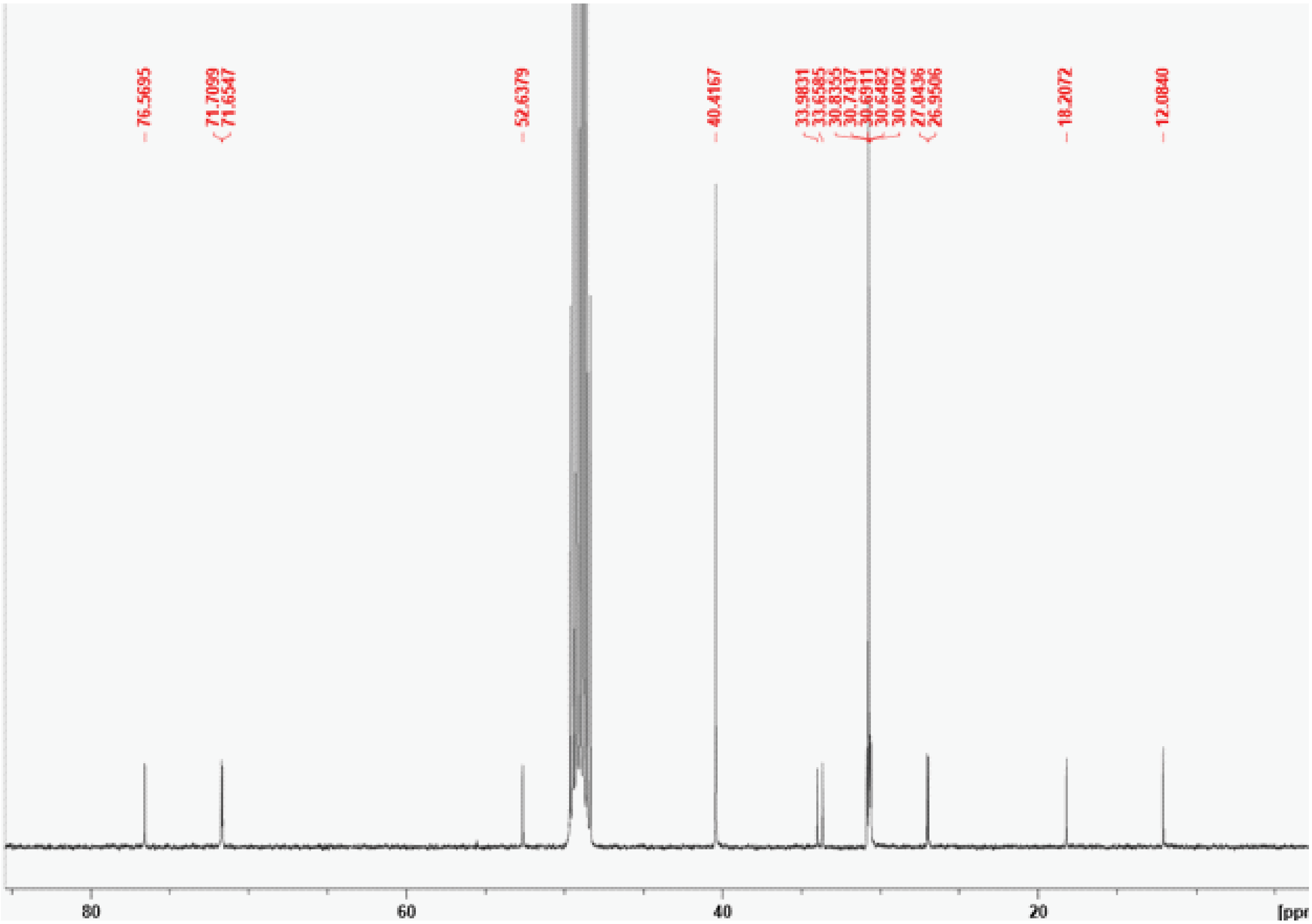
76.5695
71.7099
71.6547
52.6379
40.4167
33.9831
33.6585
30.8355
30.7437
30.6911
30.6482
30.6002
27.0436
26.9506
18.2072
12.0840
80
60
40
20
[ppm

Figure S9

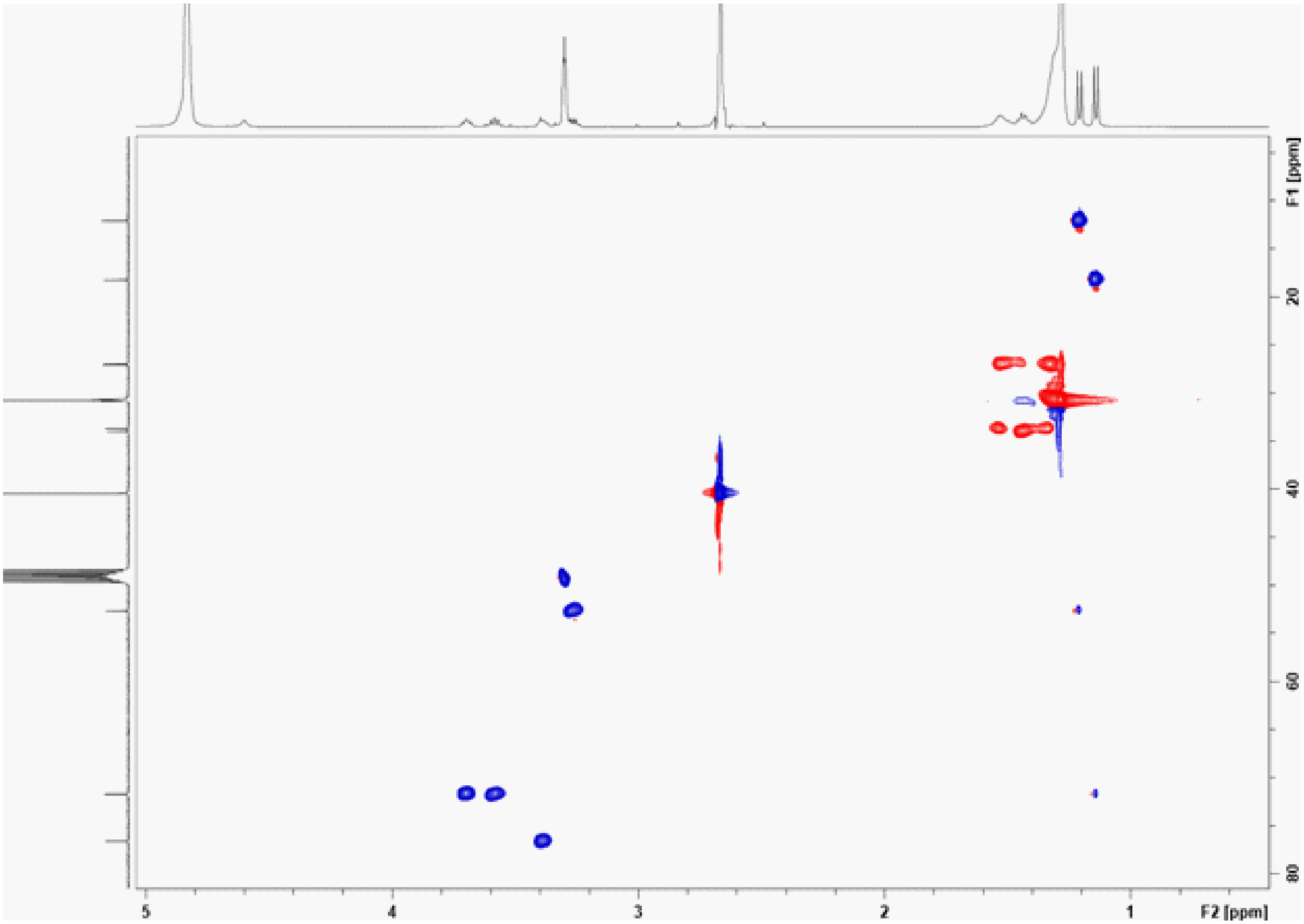

Figure S10

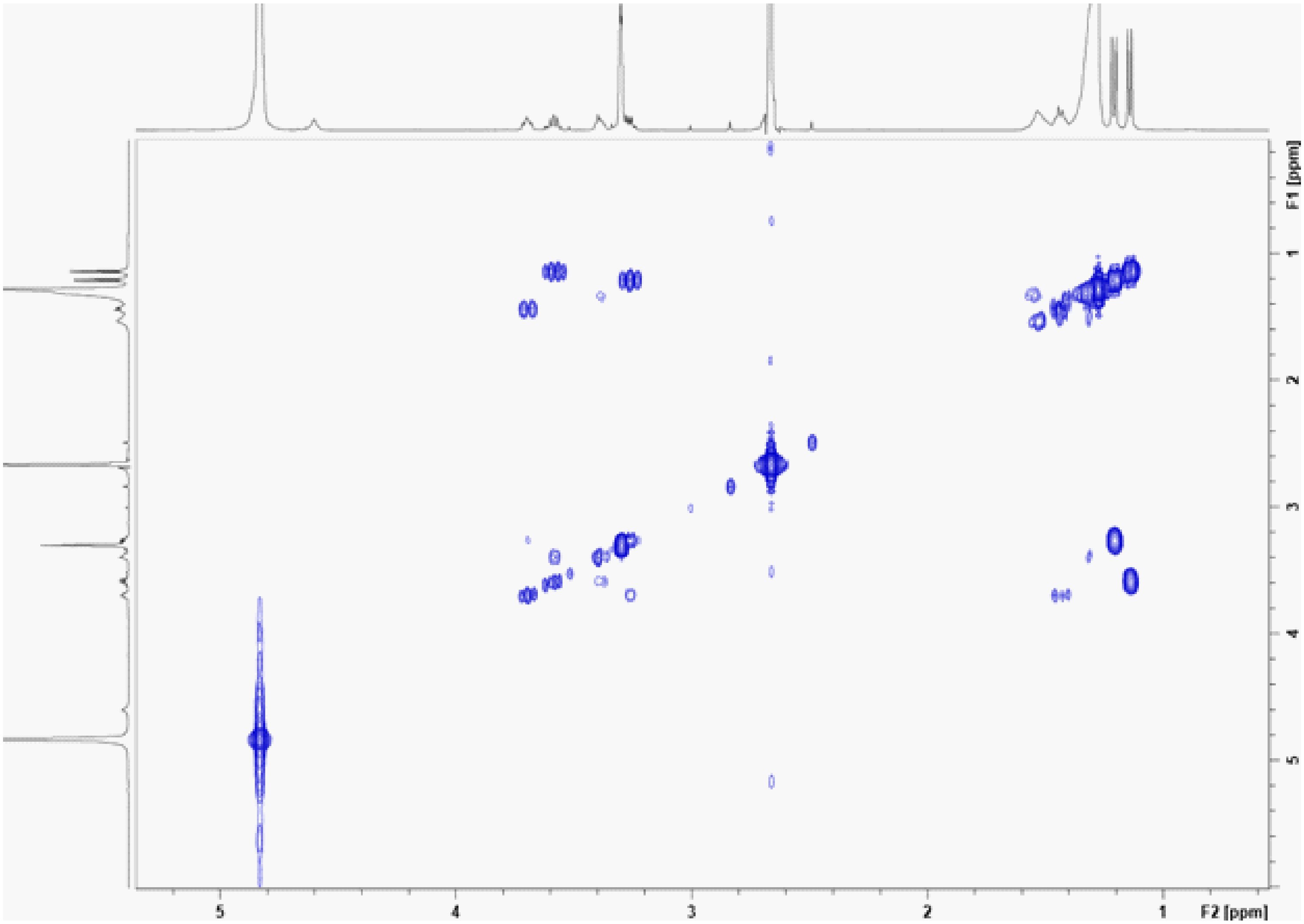

Figure S11

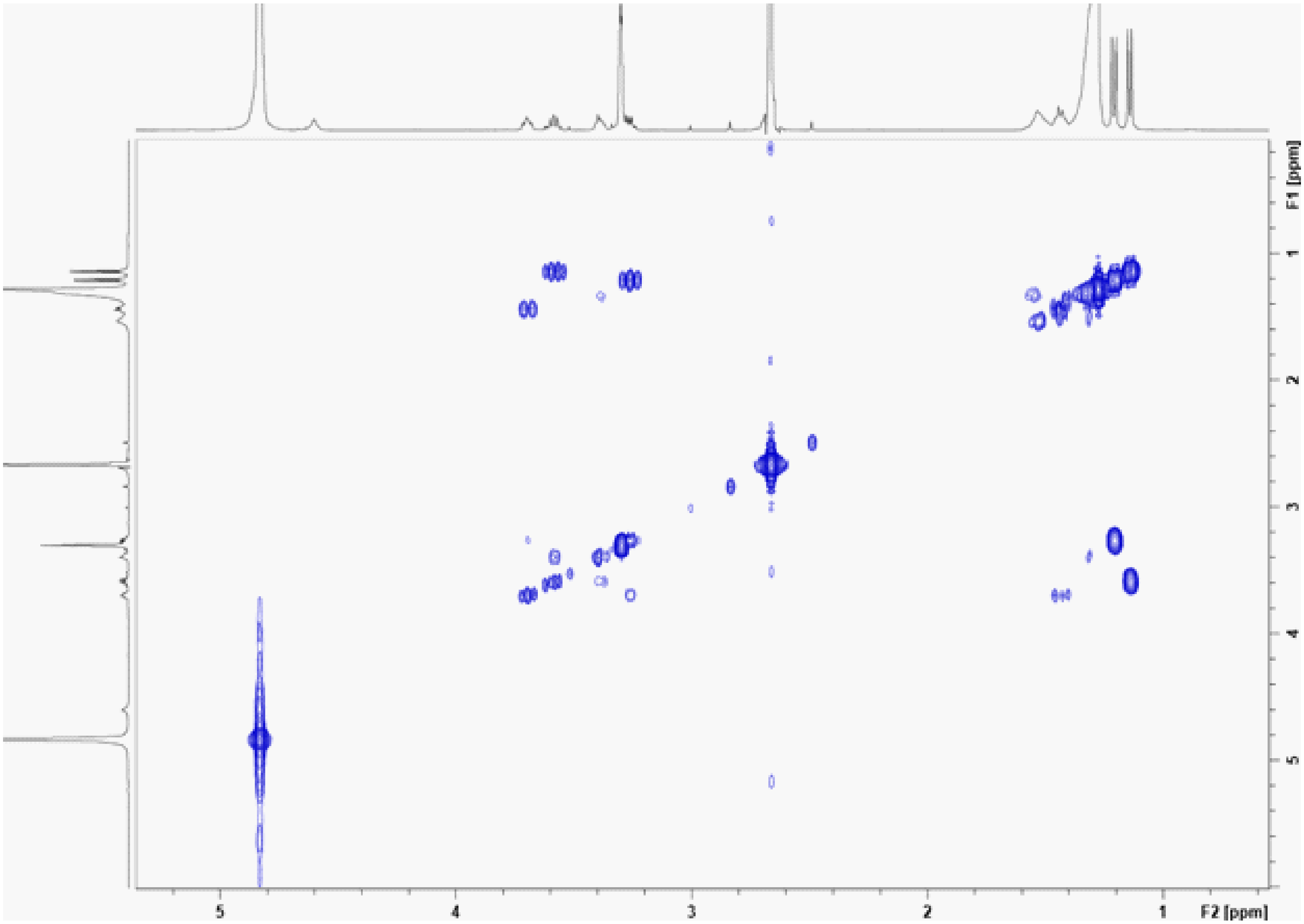

Figure S12

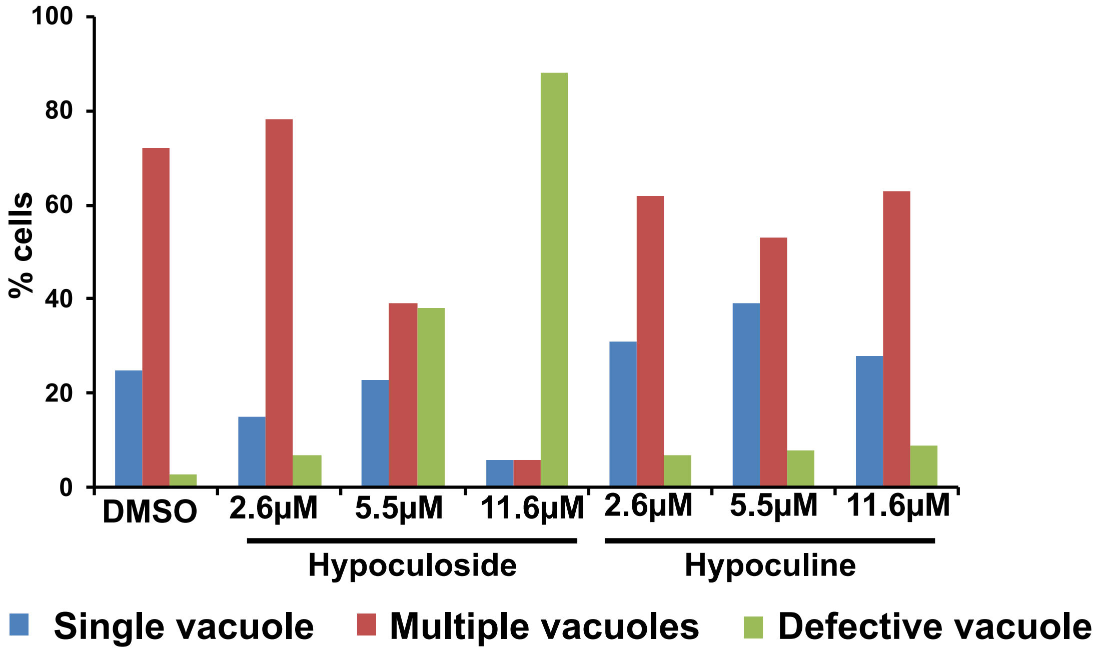
100
80
60
40
20
0
% cells
DMSO
2.6µM
5.5µM
11.6µM
2.6µM
5.5µM
11.6µM
Hypoculoside
Hypoculine
Single vacuole
Multiple vacuoles
Defective vacuole

Figure S13

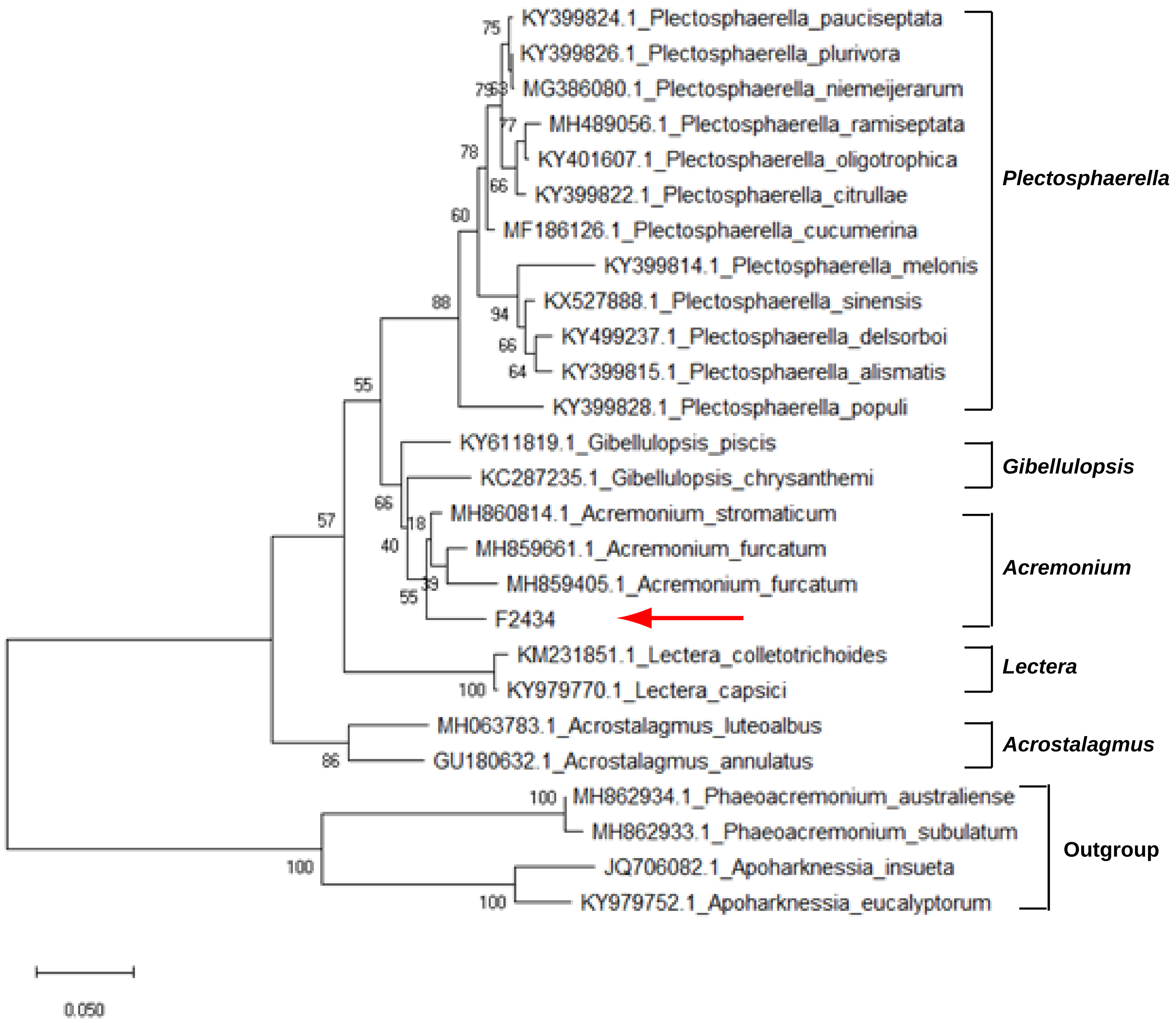
KY399824.1_Plectosphaerella_pauciseptata
KY399826.1_Plectosphaerella_plurivora
MG386080.1_Plectosphaerella_niemeijerarum
MH489056.1_Plectosphaerella_ramiseptata
KY401607.1_Plectosphaerella_oligotrophica
KY399822.1_Plectosphaerella_citrullae
MF186126.1_Plectosphaerella_cucumerina
KY399814.1_Plectosphaerella_melonis
KX527888.1_Plectosphaerella_sinensis
KY499237.1_Plectosphaerella_delsorboi
KY399815.1_Plectosphaerella_alismatis
KY399828.1_Plectosphaerella_populi
KY611819.1_Gibellulopsis_piscis
KC287235.1_Gibellulopsis_chrysanthemi
MH860814.1_Acremonium_stromaticum
MH859661.1_Acremonium_furcatum
MH859405.1_Acremonium_furcatum
F2434
KM231851.1_Lectera_colletotrichoides
KY979770.1_Lectera_capsici
MH063783.1_Acrostalagmus_luteoalbus
GU180632.1_Acrostalagmus_annulatus
MH862934.1_Phaeoacremonium_australiense
MH862933.1_Phaeoacremonium_subulatum
JQ706082.1_Apoharknessia_insueta
KY979752.1_Apoharknessia_eucalyptorum
Plectosphaerella
Gibellulopsis
Acremonium
Lectera
Acrostalagmus
Outgroup
0.050

Figure S14
